# LncRNA‐ATB regulates epithelial‐mesenchymal transition progression in pulmonary fibrosis via sponging miR‐29b‐2‐5p and miR‐34c‐3p

**DOI:** 10.1111/jcmm.16758

**Published:** 2021-06-27

**Authors:** Qi Xu, Demin Cheng, Yi Liu, Honghong Pan, Guanru Li, Ping Li, Yan Li, Wenqing Sun, Dongyu Ma, Chunhui Ni

**Affiliations:** ^1^ Key Laboratory of Modern Toxicology of Ministry of Education Center for Global Health School of Public Health Nanjing Medical University Nanjing China

**Keywords:** ceRNA, EMT, lncRNA‐ATB, pulmonary fibrosis

## Abstract

Dysregulation of non‐coding RNAs (ncRNAs) has been proved to play pivotal roles in epithelial‐mesenchymal transition (EMT) and fibrosis. We have previously demonstrated the crucial function of long non‐coding RNA (lncRNA) ATB in silica‐induced pulmonary fibrosis‐related EMT progression. However, the underlying molecular mechanism has not been fully elucidated. Here, we verified miR‐29b‐2‐5p and miR‐34c‐3p as two vital downstream targets of lncRNA‐ATB. As opposed to lncRNA‐ATB, a significant reduction of both miR‐29b‐2‐5p and miR‐34c‐3p was observed in lung epithelial cells treated with TGF‐β1 and a murine silicosis model. Overexpression miR‐29b‐2‐5p or miR‐34c‐3p inhibited EMT process and abrogated the pro‐fibrotic effects of lncRNA‐ATB in vitro. Further, the ectopic expression of miR‐29b‐2‐5p and miR‐34c‐3p with chemotherapy attenuated silica‐induced pulmonary fibrosis in vivo. Mechanistically, TGF‐β1‐induced lncRNA‐ATB accelerated EMT as a sponge of miR‐29b‐2‐5p and miR‐34c‐3p and shared miRNA response elements with MEKK2 and NOTCH2, thus relieving these two molecules from miRNA‐mediated translational repression. Interestingly, the co‐transfection of miR‐29b‐2‐5p and miR‐34c‐3p showed a synergistic suppression effect on EMT in vitro. Furthermore, the co‐expression of these two miRNAs by using adeno‐associated virus (AAV) better alleviated silica‐induced fibrogenesis than single miRNA. Approaches aiming at lncRNA‐ATB and its downstream effectors may represent new effective therapeutic strategies in pulmonary fibrosis.

## 1 INTRODUCTION

Pulmonary fibrosis may be the outcome of direct lung tissue damage caused by genetic defects and microbial agents, occupational, environmental or drug exposure.^1^ Occupational silica exposure is the main risk factor of silicosis, which is an occupational pulmonary fibrosis disease with high morbidity and mortality.^2^ Pathologically, persistent epithelial injury, epithelial‐mesenchymal transition (EMT), fibroblast activation and expansion, and extracellular matrix (ECM) accumulation are important pathological components of pulmonary fibrosis.^3^ Currently, intensive efforts are devoted to the discovery of drugs able to interfere with these pathological processes for the treatment of pulmonary fibrosis.

Epithelial‐mesenchymal transition represents a biological programme during which epithelial cells lose their identity and acquire a mesenchymal phenotype. Converging lines of evidence suggest that EMT plays a crucial role in embryonic development, cancer progression, wound healing and tissue fibrosis.^4^, ^5^ Moreover, transforming growth factor‐β1 (TGF‐β1), the major pro‐fibrotic effector, has also been proved to be a key trigger of EMT.^6^, ^7^ Following TGF‐β1 exposure, epithelial cells lose epithelial differentiation markers (E‐cadherin) and begin to produce mesenchymal markers (Vimentin).^8^ Still, inhibition of TGF‐β1 is associated with severe side effects due to its pleiotropic role. Therefore, in addition to inhibiting fibroblast activation and proliferation, identification of downstream effectors of TGF‐β1 associated with EMT might represent new therapeutic targets whose modulation may be well‐tolerated.

Approximately 98% of human genome transcripts with limited or no protein‐coding capacity are known as non‐coding RNAs (ncRNAs). Among the various types of ncRNAs, microRNA and lncRNA are receiving much attention. Emerging shreds of evidence have shown that lncRNA plays a critical role in EMT and fibrosis as a competing endogenous RNA (ceRNA) for miRNAs, thus causing the derepression of miRNA targets. For instance, lncRNA ZEB1‐AS1 enhances EMT process and BLM‐induced fibrogenesis by modulation of a miR‐141‐3p/ZEB1 feedback loop.^9^ LncRNA MALAT1 promotes EMT by regulating PI3K/AKT signalling pathway via sponging miR‐503 in silica‐induced pulmonary fibrosis.^10^ Considering that the aberrant expression of ncRNAs has a causative role in pulmonary fibrosis, it also provides a foundation for ncRNA‐based therapies.

LncRNA‐ATB, a long non‐coding RNA with a length of 2446bp, is named lncRNA‐activated by TGF‐β due to its essential role in the TGF‐β signalling pathway.^11^ The purpose of lncRNA‐ATB in cancer has been extensively studied, and several reports suggested that lncRNA‐ATB is involved in cell metastasis and invasion by regulating EMT.^12^, ^13^, ^14^ Our previous studies have reported that lncRNA‐ATB is up‐regulated in TGF‐β1‐induced A549 and BEAS‐2B cells and partially promotes EMT process by targeting miR‐200c.^15^ Of particular interest, the expression of both miR‐29b‐2‐5p and miR‐34c‐3p was also dysregulated after lncRNA‐ATB knockdown in our previous miRNAs array. However, whether these two miRNAs play a role in pulmonary fibrosis–related EMT remains largely unexplored.

In this study, we demonstrated that miR‐29b‐2‐5p and miR‐34c‐3p were two new attractive downstream targets of lncRNA‐ATB and conducted a series of experiments aiming at demonstrating how these two miRNAs mechanistically mediate the effects of lncRNA‐ATB and influence the TGF‐β1‐induced EMT process. Moreover, we also tested the benefits of miR‐29b‐2‐5p and miR‐34c‐3p overexpression in silica‐induced pulmonary fibrosis at the preclinical level and implied that pharmacological approaches aiming at lncRNA‐ATB and its downstream miRNAs may represent new effective therapeutic strategies in pulmonary fibrosis.

## 2 MATERIALS AND METHODS

### 2.1 Mouse model and ethics statement

All animal studies were conducted following human‐animal care standards, and all experimental protocols were approved by the Nanjing Medical University Ethics Committee (Nanjing, China).

Four‐week‐old male C57BL/6 mice were purchased from the Animal Core Facility of Nanjing Medical University and housed in a specific pathogen–free animal facility. Mice were randomly assigned into four groups (n = 8/group). To build a silica‐induced pulmonary fibrosis mouse model, mice under anaesthesia were intratracheally administered a single installation with 50 mg/kg of silica. Control group mice were received sham treatment with saline. The particle size was 0.5∼10 μm, and they exhibited good monodispersity. Mice were harvested on day 7, 14 and 28 after silica or saline treatment.

MiR‐29b‐2‐5p agomir and miR‐34c‐3p agomir were designed for miRNA overexpression by GenePharma Co., Ltd. 5nmol miR‐29b‐2‐5p agomir, miR‐34c‐3p agomir or control agomir was co‐administered intratracheally with silica‐suspended saline. Then, 2.5 nmol of miR‐29b‐2‐5p agomir, miR‐34c‐3p agomir or control agomir was injected via the tail vein each week. The mice were killed on day 28 after silica administration.

AAV‐miR‐29b‐2‐5p and AAV‐miR‐34c‐3p vectors were designed for miRNA overexpression by Hanbio Biotechnology Co., Ltd. Mice were administered with AAV‐miR‐29b‐2‐5p, AAV‐miR‐34c‐3p or AAV‐NC intratracheally at a dose of 1 × 10^11^ vectors in a total of 0.05 mL of sterile saline. Three weeks later, mice were treated with 50 mg/kg SiO_2_, and the control group was treated with 0.05mL of sterile saline using the same method for 4 weeks. All mice were killed, and the lungs were isolated and stored at −80℃ immediately for further analysis.

### 2.2 Cell culture and treatment

A549 and BEAS‐2B were obtained from the American Type Culture Collection (ATCC). BEAS‐2B cells were maintained in Dulbecco's modified Eagle's medium (DMEM, Life Technologies/Gibco) supplemented with 5% foetal bovine serum, 100 U/mL penicillin and 100 μg/mL streptomycin (Beyotime). A549 were maintained in RPMI‐1640 (Life Technologies/Gibco) containing 10% foetal calf serum (FCS, Life Technologies/Gibco), 100 U/mL penicillin and 100 μg/mL streptomycin (Beyotime). Cells were maintained at 37℃ and 5% CO_2_. Human primary type II alveolar epithelial cells (AECs) were obtained from Procell Biotechnology and cultured in human type II alveolar epithelial cell medium.

### 2.3 Real‐time PCR

Total RNA from cultured cells or mouse tissues was extracted and dissolved in RNase‐free water. To determine the expression of miR‐29b‐2‐5p and miR‐34c‐3p, total RNA (500 ng) was reversely transcribed with HiScript^®^ II Q Select RT SuperMix for qPCR Kit, according to the manufacturer's instructions followed by real‐time PCR, using The AceQ^®^ qPCR SYBR^®^ Green Master Mix kit (Vazyme Biotech Co., Ltd.). MiRNA expression was normalized to endogenous U6 snRNA expression. To determine lncRNA‐ATB expression, reverse transcription was performed using HiScript^®^ II Q RT SuperMix for qPCR Kit (Vazyme Biotech Co., Ltd.). Next, the AceQ^®^ qPCR SYBR^®^ Green Master Mix kit was used for lncRNA‐ATB amplification. LncRNA‐ATB expression was normalized to GAPDH expression. All qPCR primers were designed by Genery Co., Ltd., and qRT‐PCR analysis was performed using LightCycer^®^480II.

### 2.4 Western blot

Total protein lysates of tissues and cells were prepared using lysis buffer. The quantity of protein in the lysates was measured using a BCA kit (Beyotime), and equal amounts (80 μg) of proteins were separated by 10% SDS‐PAGE gel and transferred to polyvinylidene fluoride (PVDF) membranes (Millipore). After blocking with fat‐free milk for 1 hour, membranes were incubated with primary antibodies overnight at 4℃. HRP‐conjugated anti‐rabbit IgG (A0208, 1:1000, Beyotime) and HRP‐conjugated anti‐mouse IgG (H + L) (A0216, 1:1000, Beyotime) were used as secondary antibodies for 1 hour, followed by 30 minutes of washing with TBST at room temperature. Subsequently, membranes were imaged immediately using ChemiDoc XRS + (Bio‐Rad Laboratories).

### 2.5 Dual‐luciferase reporter gene assay

The wild sequence of lncRNA‐ATB, MEKK2 and NOTCH2 contained miR‐29b‐2‐5p or miR‐34c‐3p binding sites, and their mutant sequence (MUT) was chemically synthesized and inserted into pmirGLO‐Report luciferase vector (Generay Biotechnology). The luciferase reporter plasmids WT and MUT were co‐transfected into A549 cells (ATCC) with miR‐29b‐2‐5p mimic, miR‐34c‐3p mimic or control mimic. After a 24‐hour period of transfection, the cells were harvested and lysed. Luciferase activity was next detected on a luminometer TD‐20/20 detector by using a dual‐luciferase reporter assay system.

### 2.6 Immunostaining

After treatment, A549 and BEAS‐2B cells were washed twice with PBS and fixed with 4% paraformaldehyde for 30 minutes at room temperature. Cells were incubated with the antibody against E‐cadherin (1:200; Cell Signaling Technology) or the antibody against Vimentin (1:200; Cell Signaling Technology) overnight at 4℃. After washing with PBST, the cells were incubated with Cy3‐conjugated goat anti‐rabbit or FITC‐conjugated goat anti‐rabbit secondary antibodies (Beyotime) for 1 hour in dark conditions. Then, cells were then stained with DAPI (Beyotime) for 5 minutes, and immunofluorescence was observed under a fluorescence microscope (Zeiss, LSM700B).

### 2.7 RNA immunoprecipitation (RIP) assay

The RIP assay was performed using an EZ‐Magna RIP kit (Millipore) according to the manufacturer's protocol. A549 cells were collected and lysed with RIP lysis buffer containing RNase inhibitor and protease inhibitor cocktail. Then, the cell supernatants were incubated with magnetic beads conjugated with anti‐AGO2 (Abcam, No.32381) or anti‐IgG by rotating at 4℃ for 24 hours. Next, the immunoprecipitated RNA was extracted by using proteinase K and RNAse‐free DNase I to remove protein and DNA. The results were measured by qRT‐PCR.

### 2.8 Wound healing assay

A549 and BEAS‐2B cells were cultured in six‐well plates, and the cell monolayer was subsequently scratched with a 200‐μL pipette tip. Representative images of cell migration were photographed by phase‐contrast microscopy (200×) at 0 or 24 hours after injury. The width of the wound was determined with the Image Pro‐Plus program, and closed areas by migrated cells (%) were normalized to the 0 hour.

### 2.9 RNA pull‐down

MiR‐29b‐2‐5p and miR‐34c‐3p labelled with desthiobiotin were designed by GenePharma Co., Ltd. 1 × 10^7^ A549 cells were collected, and total RNA was extracted for RNA pull‐down assays. MiR‐29b‐2‐5p, miR‐34c‐3p or negative control RNA (50 pmol) was connected to the streptavidin magnetic beads (Thermo Scientific). Before the binding process, the beads were pre‐washed with 0.1 mol/L NaOH, 50 mmol/L NaCl and 100 mmol/L NaCl according to the manufacturer's protocol. Then, RNA lysates were incubated with the magnetic beads for 60 minutes at 4℃ with rotation. Then, the beads were washed twice with the 1× wash buffer (Thermo Scientific) and incubated with the elution buffer (Thermo Scientific) at 37℃ for 45 minutes. After centrifugation, the supernatant was obtained for qRT‐PCR analysis.

### 2.10 Statistical analysis

Each experiment was done at least three independent times. All results were summarized and are presented as means ± standard deviation (SD). Student's t test (unpaired, two‐tailed) was utilized to compare the means of two groups, while one‐way ANOVA (with post hoc analysis) was used to compare the means of three or more groups. *P* < .05 was considered statistically significant.

## 3 RESULTS

### 3.1 LncRNA‐ATB acts as a sponge of miR‐29b‐2‐5p and miR‐34c‐3p

**FIGURE 1:**
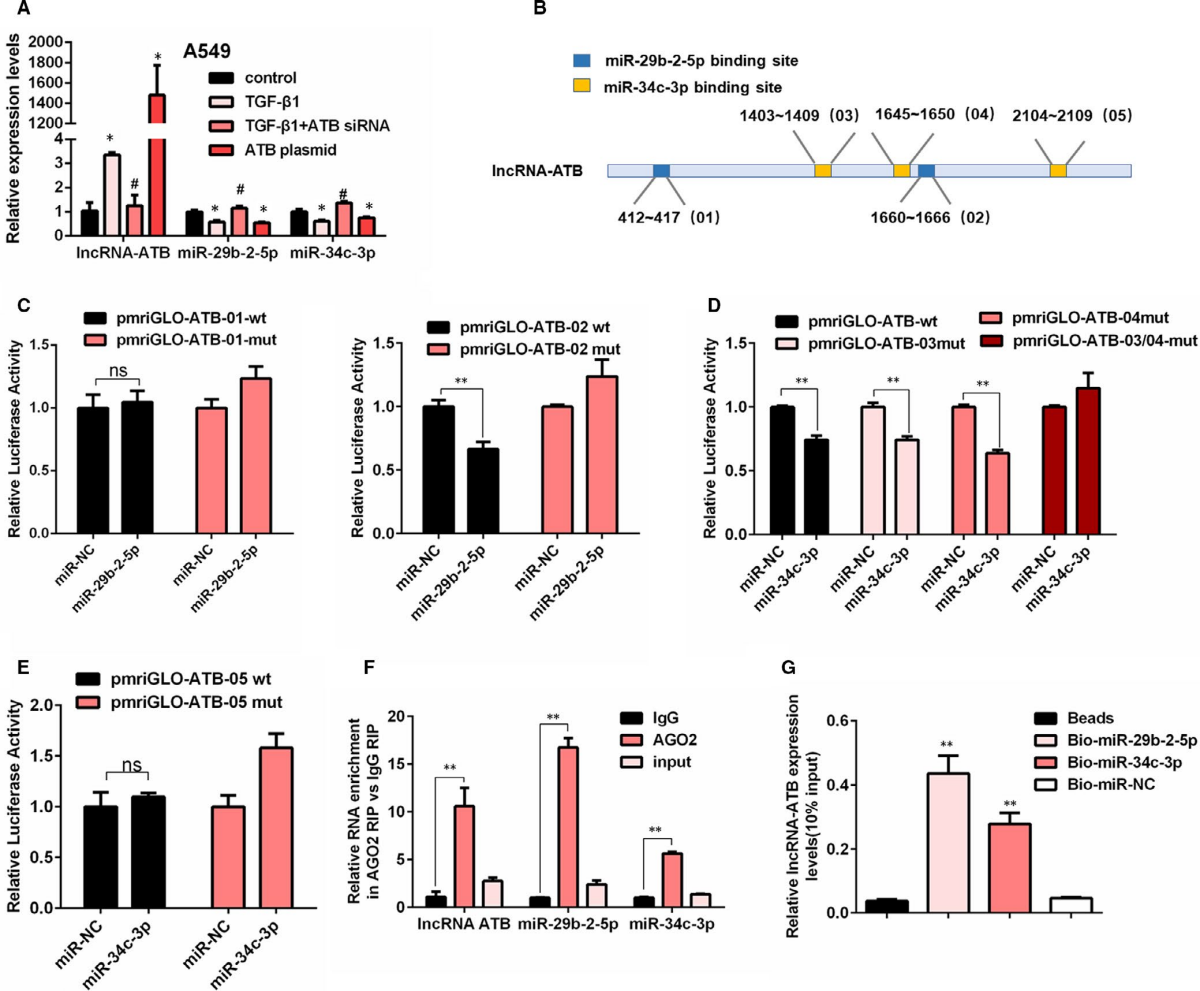
LncRNA‐ATB acts as a sponge of miR‐29b‐2‐5p and miR‐34c‐3p. A, qRT‐PCR analysis of lncRNA‐ATB, miR‐29b‐2‐5p and miR‐34c‐3p expression in A549 cells (mean ± SD, n = 3), with *P < .05 vs the control group and # *P* < .05 vs the TGF‐β1‐treated group. B, Schematic diagram of target sites of miR‐29b‐2‐5p and miR‐34c‐3p in the lncRNA‐ATB. C, Luciferase reporter assays of relative luciferase activity of in A549 transfected with ATB‐01‐wt/ATB‐01‐mut and ATB‐02‐wt/ATB‐02‐mut (mean ± SD, n = 3), with **P < .01 vs the miR‐29b‐2‐5p NC‐mimic group. D, Luciferase reporter assays of relative luciferase activity of in A549 transfected with ATB‐03/04‐wt, ATB‐03‐mut, ATB‐04‐mut and ATB‐03/04‐mut (mean ± SD, n = 3), with **P < .01 vs the miR‐34c‐3p NC‐mimic group. E, Luciferase reporter assays of relative luciferase activity of in A549 transfected with ATB‐05‐wt and ATB‐05‐mut (mean ± SD, n = 3). F, miR‐29b‐2‐5p, miR‐34c‐3p and lncRNA‐ATB enrichment as determined from RIP assays performed using IgG or AGO2 antibodies, followed by qRT‐PCR (mean ± SD, n = 3), **P < .01. G, The relative levels of lncRNA‐ATB in A549 cells were pulled down by biotinylated miR‐29b‐2‐5p or miR‐34c‐3p (mean ± SD, n = 3), with **P < .01 vs Bio‐miR‐NC group

Our previous miRNA microarray indicated that miR‐29b‐2‐5p and miR‐34c‐3p were two potential targets of lncRNA‐ATB.^15^ To further verify the microarray results, the endogenous expression of lncRNA‐ATB and two miRNAs were detected via qRT‐PCR in A549 cells. The results showed that the expression of miR‐29b‐2‐5p and miR‐34c‐3p was decreased after TGF‐β1 exposure or lncRNA‐ATB overexpression, whereas they were up‐regulated following lncRNA‐ATB knockdown (Figure 1A). As presented in Figure 1B, there were two putative binding sites for miR‐29b‐2‐5p and three potential binding sites for miR‐34c‐3p on lncRNA‐ATB. We defined two putative miR‐29b‐2‐5p binding sites as 01 and 02, and three putative miR‐34c‐3p binding sites were defined as 03, 04 and 05. Then, a luciferase assay was performed to verify the interaction between lncRNA‐ATB and miRNAs. Compared with the mutant reporter, overexpression of miR‐29b‐2‐5p suppressed the activity of ATB‐02 wild‐type vector but not the ATB‐01 wild‐type vector, indicating that miR‐29b‐2‐5p could directly target lncRNA‐ATB by binding with the 02 site (Figure 1C). Moreover, miR‐34c‐3p mimic could inhibit the activity of ATB‐03 and ATB‐04 wild‐type reporter as well as single ATB‐03 or ATB‐04 mutant reporters, and no significant changes were observed in ATB‐03/04 mutant reporters (Figure 1D). At the same time, no significant decrease was found in ATB‐05 wild‐type vector after miR‐34c‐3p mimic transfection, suggesting that the interaction between miR‐34c‐3p and lncRNA‐ATB was mediated with the 03 and 04 binding sites, but not the 05 site (Figure 1E). Next, a RIP assay was performed to further identify the ceRNA potential of lncRNA‐ATB. By qRT‐PCR, we found that the endogenous lncRNA‐ATB and two miRNAs were predominantly enriched in the AGO2 antibody group compared with the IgG group (Figure 1F). Consistently, RNA pull‐down assays also confirmed that endogenous lncRNA‐ATB could be pulled down by biotin‐labelled miR‐29b‐2‐5p or miR‐34c‐3p (Figure 1G). These results strongly proved that lncRNA‐ATB could interact and bind with miR‐29b‐2‐5p and miR‐34c‐3p through AGO2 as a ceRNA.

### 3.2 TGF‐β1 promotes EMT process and decreases miR‐29b‐2‐5p and miR‐34c‐3p levels

**FIGURE 2:**
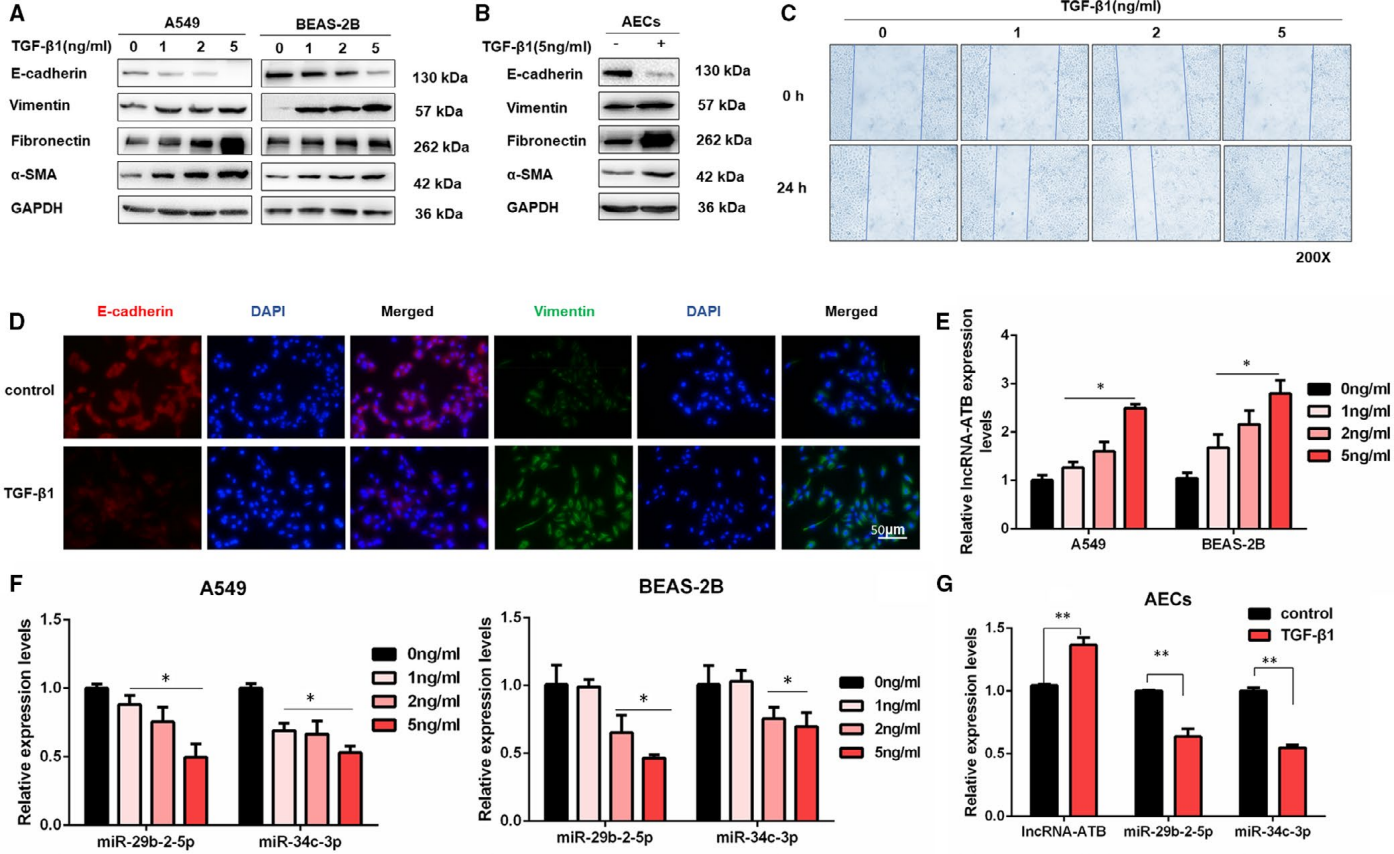
TGF‐β1 promotes EMT process and decreases miR‐29b‐2‐5p and miR‐34c‐3p levels. A, Western blot analysis of E‐cadherin, Vimentin, Fibronectin and α‐SMA in A549 cells and BEAS‐2B cells treated with 0, 1, 2 and 5 ng/mL TGF‐β1 for 48 h. B, Western blot analysis of E‐cadherin, Vimentin, Fibronectin and α‐SMA in human primary type II AECs treated with 0 or 5 ng/mL TGF‐β1 for 48 h. C, Wound healing assays were performed to measure the migration ability of A549 cells treated with 0, 1, 2 and 5 ng/mL TGF‐β1 for 48 h. D, Immunofluorescence staining of Vimentin and E‐cadherin in A549 cells for the control and TGF‐β1 (0 or 5 ng/mL) treatment groups. Green represents Vimentin staining; red represents E‐cadherin staining; and blue represents nuclear DNA staining by DAPI. The scale bar is 50 μm. E, qRT‐PCR detection of lncRNA‐ATB expression in 0, 1, 2 and 5 ng/mL TGF‐β1 treated A549 and BEAS‐2B cells for 48 h (mean ± SD, n = 3), *P < .05 difference from untreated cells. F, qRT‐PCR analysis of miR‐29b‐2‐5p and miR‐34c‐3p expression in 0, 1, 2 and 5 ng/mL TGF‐β1 treated A549 and BEAS‐2B cells for 48 h (mean ± SD, n = 3), *P < .05 difference from untreated cells. G, qRT‐PCR detection of lncRNA‐ATB, miR‐29b‐2‐5p and miR‐34c‐3p expression in human primary type II AECs after 0 or 5 ng/mL TGF‐β1 treatment (mean ± SD, n = 3), *P < .05

To confirm whether miR‐29b‐2‐5p and miR‐34c‐3p were involved in the lncRNA‐ATB‐mediated EMT regulation network, two cell lines (A549 and BEAS‐2B) and human primary type II alveolar epithelial cells (AECs) were used for the subsequent experiments. To identify TGF‐β1 induced EMT, we treated A549 and BEAS‐2B cells with TGF‐β1 at various concentrations (0, 1, 2, 5 ng/mL) for 48 hours. The down‐regulation of E‐cadherin and up‐regulation of Vimentin, Fibronectin and α‐SMA were shown to be dose‐dependent regarding TGF‐β1 treatment (Figure 2A and Figure S1A), which was also supported by human primary type II AECs (Figure 2B). Besides, wound healing assays showed that TGF‐β1 also increased the migration ability of A549 and BEAS‐2B cells (Figure 2C, Figure S1B, C). Moreover, immunofluorescence staining for E‐cadherin and Vimentin in A549 and BEAS‐2B cells revealed that 5 ng/mL TGF‐β1 reduced E‐cadherin expression and induced Vimentin expression (Figure 2D and Figure S1D). Exposure to TGF‐β1 increased lncRNA‐ATB expression while reducing the expression of miR‐29b‐2‐5p and miR‐34c‐3p in two cell lines and human primary type II AECs (Figure 2E, F, G), suggesting miR‐29b‐5p and miR‐34c‐3p could be involved in the TGF‐β1‐induced EMT process. As the isolation and culture of human primary type II AECs are complex and the cell passage is limited, A549 cell line, adenocarcinomic human alveolar basal epithelial cells possessed the characteristics of AECs, has been used in many studies for the replacement of human type II AECs. Therefore, two cell lines (A549 and BEAS‐2B) were used to explore further molecular mechanisms in the present study.

### 3.3 MiR‐29b‐2‐5p and miR‐34c‐3p mediate the function of lncRNA‐ATB in regulating EMT

**FIGURE 3:**
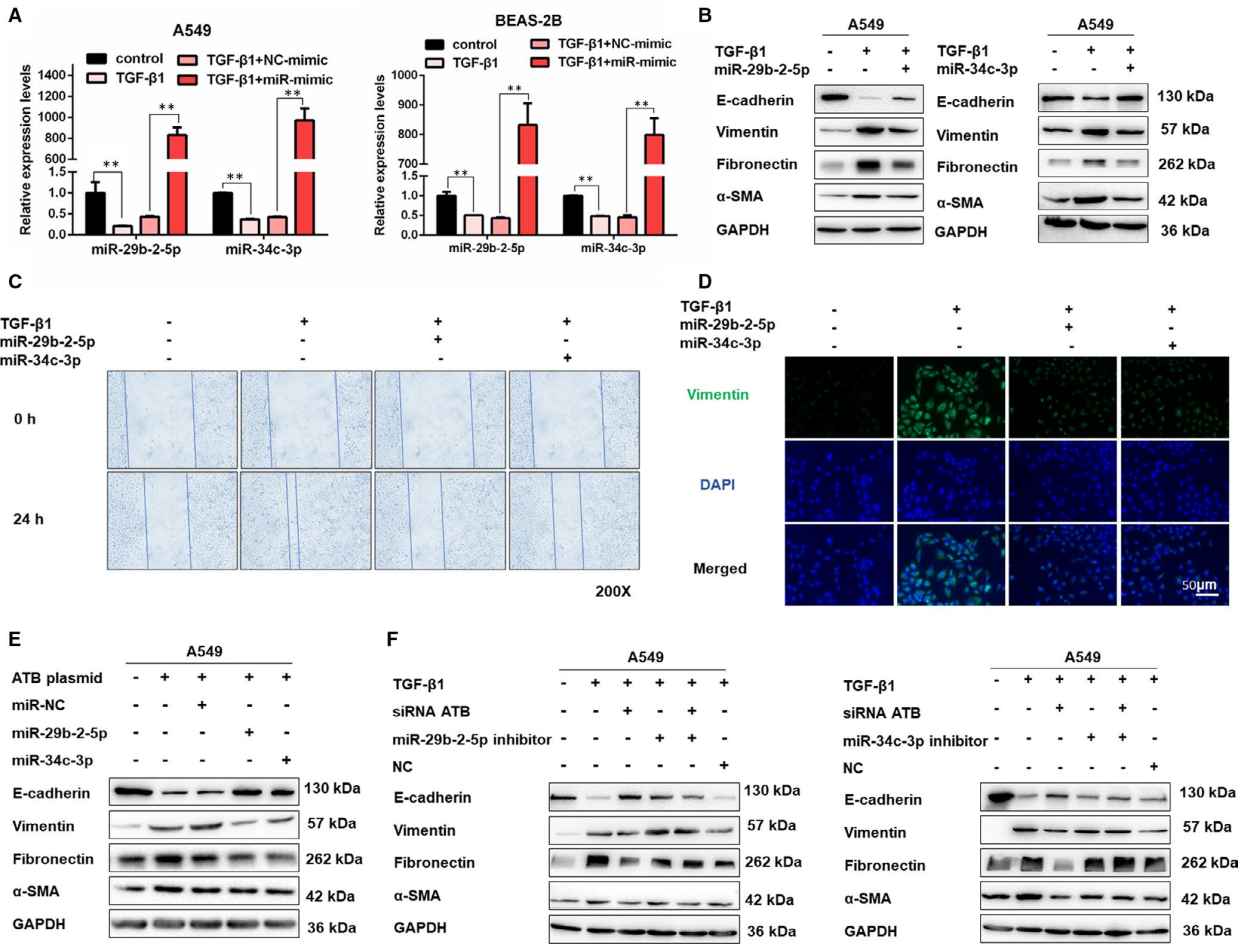
MiR‐29b‐2‐5p and miR‐34c‐3p mediate the function of lncRNA‐ATB in regulating EMT. A, The miR‐29b‐2‐5p and miR‐34c‐3p were detected by qRT‐PCR in A549 and BEAS‐2B cells transfected with miR‐29b‐2‐5p mimic, miR‐34c‐3p mimic or NC‐mimic (mean ± SD, n = 3), **P < .01. B, Western blot detected levels of E‐cadherin, Vimentin, Fibronectin and α‐SMA in A549 cells transfected with miR‐29b‐2‐5p or miR‐34c‐3p mimic then treated with 5 ng/mL TGF‐β1 for 48 h. C, Cell migration was measured using a wound healing assay in A549 cells transfected with miR‐29b‐2‐5p or miR‐34c‐3p mimic then treated with 5 ng/mL TGF‐β1 for 48 h (mean ± SD, n = 3), **P < .05 difference from untreated cells. D, The expression of Vimentin was detected by immunofluorescence staining in A549 cells transfected with miR‐29b‐2‐5p or miR‐34c‐3p mimic then treated with 5 ng/mL TGF‐β1 for 48 h. E‐F, Western blot of the protein expression of E‐cadherin, Vimentin, Fibronectin and α‐SMA in treated A549 cells for the indicated groups

To further confirm the functional role of miR‐29b‐2‐5p and miR‐34c‐3p, we overexpressed miR‐29b‐2‐5p and miR‐34c‐3p by using miRNA mimic in A549 and BEAS‐2B cell lines (Figure 3A). As indicated by the Western blot results, both miR‐29b‐2‐5p and miR‐34c‐3p ectopic expression inhibited Vimentin, Fibronectin and α‐SMA expression and reversed the suppression of E‐cadherin by TGF‐β1 (Figure 3B and Figure S2A). Accordingly, the migration activity of A549 and BEAS‐2B cells induced by TGF‐β1 was also decreased after miR‐29b‐2‐5p or miR‐34c‐3p overexpression (Figure 3C, Figure S2B, C). As shown in the immunostaining analysis, the fluorescence of Vimentin was markedly weaker in miR‐29b‐2‐5p and miR‐34c‐3p transfected cells (Figure 3D and Figure S2D). Besides, the overexpressed lncRNA‐ATB could induce EMT, whereas combined incubated with miR‐29b‐2‐5p or miR‐34c‐3p mimics abrogated the effects of lncRNA‐ATB (Figure 3E). In contrast, lncRNA‐ATB knockdown effectively suppressed the EMT process, and its function was reversed by miR‐29b‐2‐5p and miR‐34c‐3p inhibitors (Figure 3F, Figure S2E, F). These results suggested that miR‐29b‐2‐5p and miR‐34c‐3p mediated the function of lncRNA‐ATB in regulating EMT.

### 3.4 MEKK2 and NOTCH2 are two functional downstream targets of miR‐29b‐2‐5p and miR‐34c‐3p

**FIGURE 4:**
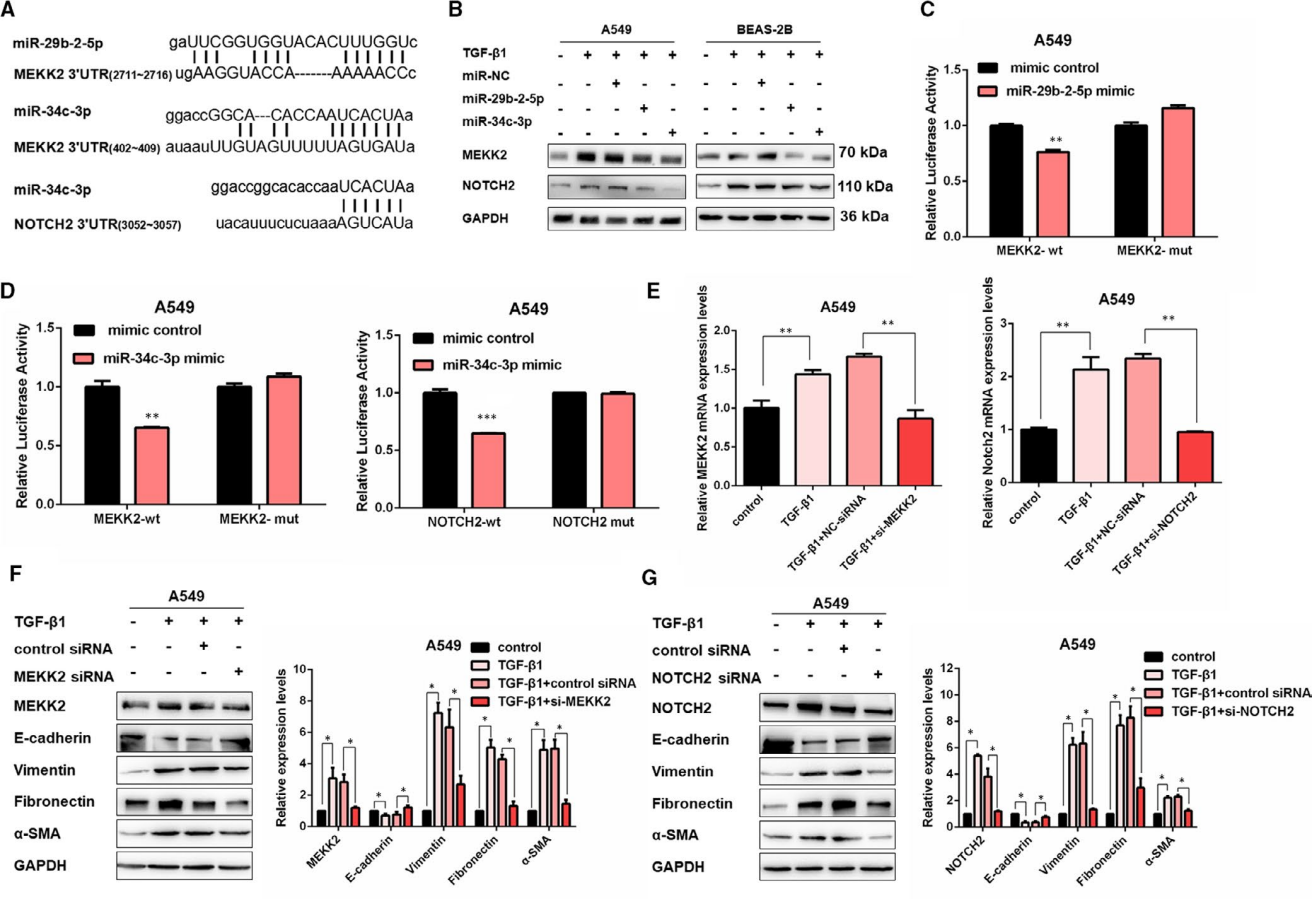
MEKK2 and NOTCH2 are two functional downstream targets of miR‐29b‐2‐5p and miR‐34c‐3p. A, Schematic diagram of conserved target sites of miR‐29b‐2‐5p in the 3'UTR of MEKK2 mRNA and miR‐34c‐3p in the 3'‐UTR of MEKK2 mRNA and NOTCH2 mRNA. B, Western blot detected levels of E‐cadherin, Vimentin, Fibronectin and α‐SMA in A549 and BEAS‐2B cells for the indicated groups. C, Luciferase reporter assays of relative luciferase activity of in A549 transfected with MEKK2‐wt or MEKK2‐mut (mean ± SD, n = 3), with **P < .01 vs the NC‐mimic group. D, Luciferase reporter assays of relative luciferase activity of in A549 transfected with MEKK2‐wt/MEKK2‐mut or NOTCH2‐wt /NOTCH2‐mut (mean ± SD, n = 3), with **P < .01 and ***P < .01 vs the NC‐mimic group. E, qRT‐PCR analysis of MEKK2 and NOTCH2 mRNA expression in A549 cells transfected with MEKK2 siRNA, NOTCH2 siRNA or NC‐siRNA (mean ± SD, n = 3), **P < .01. F, Western blot and densitometric analysis of MEKK2, E‐cadherin, Vimentin, Fibronectin, and α‐SMA in A549 cells transfected with MEKK2 siRNA or its negative control then treated with 5 ng/mL TGF‐β1 for 48 h (mean ± SD, n = 3), *P < .05. G, Western blot and densitometric analysis of NOTCH2, E‐cadherin, Vimentin, Fibronectin and α‐SMA in A549 cells transfected with NOTCH2 siRNA or its negative control then treated with 5 ng/mL TGF‐β1 for 48 h (mean ± SD, n = 3), *P < .05

By searching the miRNA databases (miRbase, TargetScan and miRDB), one binding site for miR‐29b‐2‐5p and another binding site for miR‐34c‐3p in the MEKK2 mRNA 3'‐untranslated region (3'‐UTR) were observed. Moreover, one binding site for miR‐34c‐3p was also confirmed in the NOTCH2 mRNA 3' ‐UTR (Figure 4A). It is reported that both MEKK2 and NOTCH2 contribute to the EMT process.^16^, ^17^, ^18^ Consistently, our data also demonstrated MEKK2 and NOTCH2 strikingly up‐regulated in TGF‐β1‐stimulated A549 and BEAS‐2B cells (Figure S3A). Furthermore, ectopic miR‐29b‐2‐5p and miR‐34c‐3p expression abrogated the increase of MEKK2 and NOTCH2 induced by TGF‐β1 at the protein level (Figure 4B). Accordingly, up‐regulated E‐cadherin and down‐regulated mesenchymal markers were observed in cells transfected with miR‐29b‐2‐5p or miR‐34c‐3p mimic (Figure S3B). Moreover, miR‐29b‐2‐5p mimic reduced the luciferase activity of the MEKK2‐wt reporter but did not affect the mutant reporter (Figure 4C). As expected, the luciferase activity of MEKK2‐wt and NOTCH2‐wt reporter genes decreased when miR‐34c‐3p mimic was transfected (Figure 4D). Then, successful knockdown of MEKK2 or NOTCH2 using siRNAs resulted in up‐regulated E‐cadherin and down‐regulated Vimentin at the protein levels, which was similar to miR‐29b‐2‐5p or miR‐34c‐3p overexpression (Figure 4E‐G, Figure S3C, D). All of these results revealed the important role of miR‐29b‐2‐5p and miR‐34c‐3p in EMT by targeting MEKK2 and NOTCH2.

### 3.5 MiR‐29b‐2‐5p or miR‐34c‐3p accelerates silica‐induced pulmonary fibrosis resolution by regulating EMT

**FIGURE 5:**
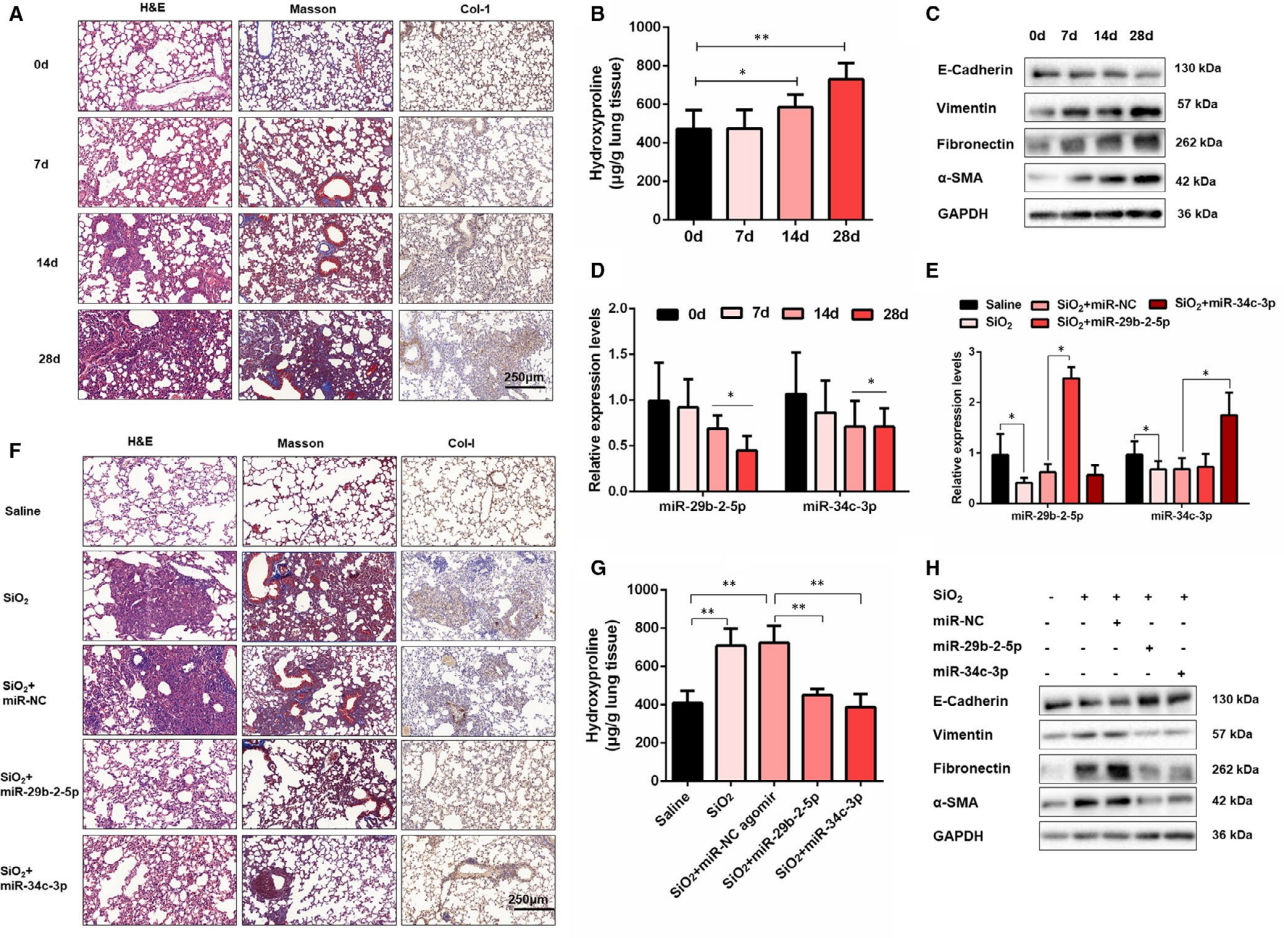
MiR‐29b‐2‐5p or miR‐34c‐3p accelerates silica‐induced pulmonary fibrosis resolution by regulating EMT. A, Histological changes and collagen deposition in lung tissues were measured by haematoxylin and eosin (H&E) staining, Masson trichrome staining and IHC staining of collagen I. B, Hydroxyproline content of the lung tissues was used to assess the degree of collagen deposition. C, Western blot analysis of the protein expression of E‐cadherin, Vimentin, Fibronectin and α‐SMA in mouse lung tissues. D, qRT‐PCR analysis of miR‐29b‐2‐5p or miR‐34c‐3p expression in mouse fibrotic lung tissue on days 7, 14 and 28, U6 was used as an internal control, with *P < .05 vs the saline group. E, qRT‐PCR analysis of miR‐29b‐2‐5p and miR‐34c‐3p expression in mouse fibrotic lung tissue on control, SiO_2_, SiO_2_ + NC‐agomir, SiO_2_ + miR‐29b‐2‐5p agomir, and SiO_2_ + miR‐34c‐3p agomir groups, *P < .05. F, The histology of the lung lesions was observed with haematoxylin and eosin (H&E) staining, Masson trichrome staining and IHC staining of collagen I. G, Hydroxyproline content of the lung tissues was used to assess the degree of collagen deposition. H, The protein expression of E‐cadherin, Vimentin, Fibronectin and α‐SMA in mouse lung tissues treated with miR‐29b‐2‐5p or miR‐34c‐3p agomir for 28 d were determined by Western blot

We next established a silica‐induced pulmonary fibrosis mouse model to examine the in vivo role of miR‐29b‐2‐5p and miR‐34c‐3p in the pulmonary fibrogenesis. Mice under anaesthesia were intratracheally administered a single installation with 50 mg/kg of silica and were harvested on day 7, 14 and 28 (Figure S4A). Histologically, the typical alveolar architecture was destroyed, and mature fibrotic nodules formed after silica exposure for 28 days. Besides, Masson's trichrome staining and IHC staining to collagen I indicated the excessive ECM deposition in the mouse lungs on day 28 (Figure 5A), and the result was further confirmed by hydroxyproline analysis (Figure 5B). Silica injury also decreased E‐cadherin expression and increased the expression of Vimentin, α‐SMA and Fibronectin (Figure 5C and Figure S4B). Then, we detected miRNAs expression in this model and observed that miR‐29b‐2‐5p and miR‐34c‐3p were markedly decreased on days 14 and 28 compared with the saline group (Figure 5D). Due to the poor conservation of lncRNA‐ATB, we did not detect its expression level in mouse lung tissue. However, compared with the control group, an increase of lncRNA‐ATB and a decrease of these two miRNAs were also observed in lung tissues of idiopathic pulmonary fibrosis (IPF) patients (Figure S4C). These data suggested that lncRNA‐ATB and its downstream miRNAs could have the potential function during pulmonary fibrosis.

Then, to confirm the role of miR‐29b‐5p and miR‐34c‐3p during silica‐induced pulmonary fibrosis, we designed miRNA‐agomir for miR‐29b‐2‐5p and miR‐34c‐3p overexpression in mice (Figure S4D). As expected, miRNA‐agomir intratracheally administration significantly elevated miR‐29b‐2‐5p or miR‐34c‐3p levels in the lung tissues of mice (Figure 5E). Histologically, extensive tissue fibrosis was observed after silica injury, whereas both miR‐29b‐2‐5p and miR‐34c‐3p overexpression exceedingly attenuated pulmonary fibrosis (Figure 5F). Moreover, as assessed by both hydroxyproline and Western blot analysis, miR‐29b‐2‐5p or miR‐34c‐3p reduced collagen deposition in the lung and decreased pro‐fibrotic mediator production at protein levels (Figure 5G, H and Figure S4E). Thus, all of these data implied that miR‐29b‐2‐5p and miR‐34c‐3p could partially restore lung structure in silica‐induced pulmonary fibrosis.

### 3.6 Combination of miR‐29b‐2‐5p and miR‐34c‐3p exerts a synergic effect on EMT and silica‐induced pulmonary fibrosis

**FIGURE 6:**
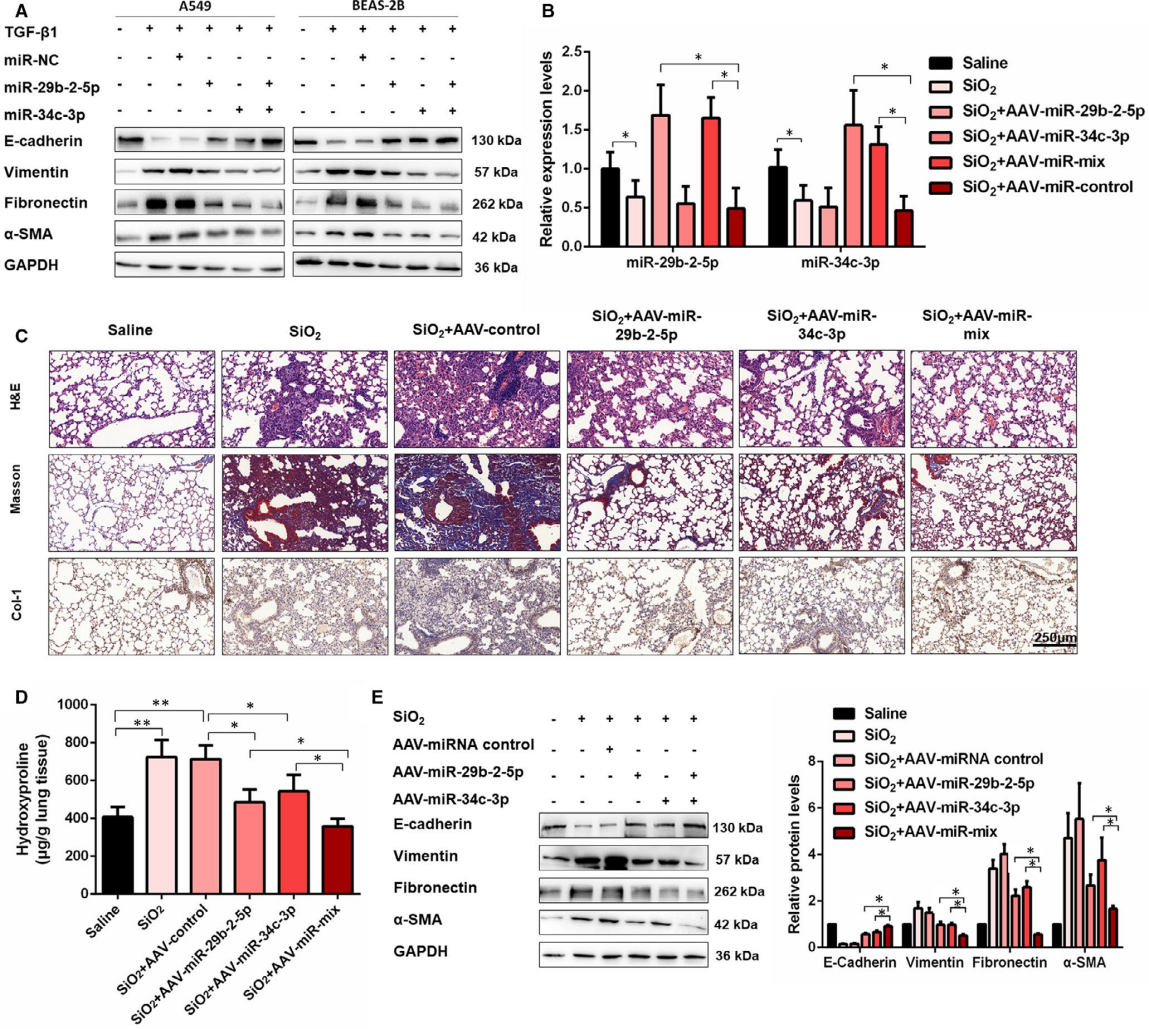
Combination of miR‐29b‐2‐5p and miR‐34c‐3p exerts a synergic effect on EMT and silica‐induced pulmonary fibrosis. A, Western blot detected levels of E‐cadherin, Vimentin, Fibronectin and α‐SMA in A549 and BEAS‐2B cells for the indicated groups. B, qRT‐PCR analysis of miR‐29b‐2‐5p and miR‐34c‐3p expression in mouse fibrotic lung tissue on saline, SiO_2_, SiO_2_ + AAV‐control, SiO_2_ + AAV‐miR‐29b‐2‐5p, SiO_2_ + AAV‐miR‐34c‐3p and SiO_2_ + AAV‐miR‐mix, with *P < .05 vs the saline group. C, The histology of the lung lesions was observed with haematoxylin and eosin (H&E) staining, Masson trichrome staining and IHC staining of collagen I. D, Hydroxyproline content of the lung tissues was used to assess the degree of collagen deposition. E, Western blot and densitometric analysis of E‐cadherin, Vimentin, Fibronectin and α‐SMA in mouse lung tissues treated with saline, SiO_2_, SiO_2_ + AAV‐control, SiO_2_ + AAV‐miR‐29b‐2‐5p, SiO_2_ + AAV‐miR‐34c‐3p and SiO_2_ + AAV‐miR‐mix (mean ± SD, n = 3)

Interestingly, we also observed that co‐expression of miR‐29b‐2‐5p and miR‐34c‐3p exerted a more significant suppression of the TGF‐β1‐induced EMT process than single miRNA in vitro (Figure 6A and Figure S5A). Moreover, immunofluorescence staining of Vimentin in A549 and BEAS‐2B cells further confirmed the synergistic effects of miR‐29b‐2‐5p and miR‐34c‐3p (Figure S5B, C). To test the synergistic effects of miR‐29b‐2‐5p and miR‐34c‐3p in vivo, we again used the silica‐induced pulmonary mouse model. AAV‐miR‐29b‐5p and AAV‐miR‐34c‐3p were designed and administrated intratracheally with mice to overexpress these two miRNAs in vivo (Figure S5D). As expected, qRT‐PCR assays demonstrated that both two miRNAs were up‐regulated in mice lung tissues by AAV induction (Figure 6B). Histologically, compared with single miRNA overexpression, co‐expression of miR‐29b‐2‐5p and miR‐34c‐3p exceedingly attenuated pulmonary fibrosis and collagen deposition, which was further supported by the results of hydroxyproline analysis (Figure 6C, D). Accordingly, co‐overexpression of two miRNAs more significantly increased E‐cadherin expression and decreased pro‐fibrotic mediators (Vimentin, Fibronectin and α‐SMA) expression than single miRNA (Figure 6E). These results demonstrated that the combination of miR‐29b‐2‐5p and miR‐34c‐3p exerts a synergic effect on EMT and silica‐induced pulmonary fibrosis.

## 4 DISCUSSION

Pulmonary fibrosis is characterized by an abnormal fibrotic response involving vast areas of the lungs. Given the poor knowledge of mechanisms underlying pulmonary fibrosis, therapeutic options are limited. Previously, we identified the lncRNA‐ATB could function as a critical regulator of TGF‐β1‐induced EMT by targeting miR‐200c during pulmonary fibrosis.^15^ Nevertheless, whether lncRNA‐ATB exerts additional pro‐fibrotic function besides targeting miR‐200c remains to be investigated. In this study, we demonstrated that miR‐29b‐2‐5p and miR‐34c‐3p were also involved in the regulatory network of lncRNA‐ATB, which further elucidated the mechanism of lncRNA‐ATB in TGF‐β1‐induced EMT process, thus providing support for its use as a potential target of fibrosis.

A variety of studies, including ours, have provided strong evidence that miRNAs play essential roles in fibrosis diseases, and many lncRNAs can act as ceRNA to regulated gene expression by binding with miRNAs, including lncRNA‐ATB. For example, lncRNA‐ATB has been proved to enforce β‐catenin expression by competitively binding the miR‐200a and subsequently promotes the activation of LX‐2 cells and hepatic fibrosis progression.^19^ Another study demonstrated that lncRNA‐ATB acts as a ceRNA to facilitate the initiation and progression of keloids by targeting miR‐200c to increase ZNF‐217.^20^ According to bioinformatics analysis, we predicted that the lncRNA‐ATB transcript contained the binding site of miR‐29b‐2‐5p and miR‐34c‐3p, suggesting they were putative targets of lncRNA‐ATB. Moreover, the expression of miR‐29b‐2‐5p and miR‐34c‐3p was decreased following TGF‐β1 exposure and enhanced after lncRNA‐ATB knockdown. Then, we demonstrated that lncRNA‐ATB could bind to these two miRNAs through dual‐luciferase reporter assays, RIP assays and RNA pull‐down assays. Taken together, our data confirmed that miR‐29b‐2‐5p and miR‐34c‐3p were two downstream targets of lncRNA‐ATB.

A growing body of research has demonstrated that both miR‐29b‐2‐5p and miR‐34c‐3p play key roles in various types of cancers.^21^, ^22^ However, little is known about these functions in pulmonary fibrosis. Only one study has addressed that miR‐29b‐2‐5p is down‐regulated in bleomycin‐induced fibrotic murine lung tissues and seems to participate in TGF‐β1‐induced lung fibroblast activation.^23^ Another study reported that the expression of miR‐34c‐3p was reduced in the lung tissues of rats and A549 cells exposed to silica.^24^ Consistently, the present study also suggested that miR‐29b‐2‐5p and miR‐34c‐3p were involved in pulmonary fibrosis and expanded their function in the EMT process. Down‐regulated miR‐29b‐2‐5p and miR‐34c‐3p were observed in A549 cells, BEAS‐2B cells and human primary type II AECs following TGF‐β1 treatment as well as in the lung upon silica treatment. Overexpression of miR‐29b‐2‐5p and miR‐34c‐3p blocked the promotion effects of TGF‐β1 on EMT process in vitro and attenuated mouse pulmonary fibrosis in vivo.

It is well characterized that miRNAs play their biological roles mainly by binding to the 3′‐UTR of the target genes. Therefore, we predicted the target genes by the bioinformatics tools and found that MEKK2 was a putative target of both miR‐29b‐2‐5p and miR‐34c‐3p, and NOTCH2 was identified as another downstream target of miR‐34c‐3p. Several studies suggest that MEKK2 can contribute to the growth and migration of cancer cells by modulating the EMT process in multiple tumour diseases, including lung cancer, breast cancer and gastric cancer.^16^, ^17^, ^25^ Besides, NOTCH2 has been demonstrated to play a key role in renal, liver and pulmonary fibrosis diseases through regulating EMT.^26^, ^27^, ^28^ Similarly, our findings further confirmed that the expression of MEKK2 and NOTCH2 was increased in A549 and BEAS‐2B cells after TGF‐β1 treatment and exerted the pro‐fibrotic effects by promoting the EMT process. These investigations indicated that miRNAs could interact with the same or different genes thus forming a complex miRNA regulatory network in diseases.

Recently, several studies have shown that combined with two or several miRNAs seems to exert a synergistic effect on the repression of downstream targets. It has been reported that the combined treatment with miR‐129 and miR‐335 induced a synergistic effect on Sp1 suppression and MMP‐9 reduction, thereby exerting potential therapeutic benefits in diabetic wound healing.^29^ Also, another study suggested that both partners of the miR‐144/451 cluster mediate a protective role in cardiomyocytes, and overexpression of the entire miR‐144/451 cluster resulted in cardioprotection to a synergistic extent.^30^ Likewise, our results showed that overexpression of miR‐29b‐2‐5p or miR‐34c‐3p could inhibit TGF‐β1 induced EMT and attenuate silica‐induced pulmonary fibrosis, and combined with these two miRNAs exerted a synergistic effect in vitro and in vivo. These data suggested that a combination of miRNAs might obtain a better therapeutic effect compared with a single miRNA.

In recent years, whether epithelial cells are transformed into myofibroblasts through the EMT process is controversial. By following the fate of type II AECs in a bleomycin‐induced mouse model of pulmonary fibrosis, Rock et al presented that type II AECs did not convert into myofibroblasts.^31^ However, it must be highlighted that the demonstration of pathobiological processes in bleomycin‐induced murine models of pulmonary fibrosis may not fit human pulmonary fibrosis. Moreover, mesenchymal‐derived cells in IPF are often found to co‐express epithelial and mesenchymal markers, denoting an incomplete transition.^32^, ^33^ This partial EMT hypothesis is also supported by Marmai's research in lung fibrosis, in which a subset of epithelial cells of patients with IPF expresses both epithelial and mesenchymal markers.^34^ Further study suggested a potential role of type II AECs that activated epithelial cells may produce mesenchymal proteins to promote activation of fibroblasts.^35^ Besides, convincing evidence has recently been provided that ZEB1‐mediated EMT in human type II AECs contributes to the development of lung fibrosis by paracrine signalling to underlying fibroblasts, suggesting that the EMT process of epithelial cells may create a profibrogenic microenvironment to contribute fibrosis progression rather than convert into mesenchymal cells directly.^36^ Our results also support the hypothesis by using two different pulmonary cell lines (A549 and BEAS‐2B) and human primary type II AECs and suggested that targeting the EMT process could be a beneficial therapy for pulmonary fibrosis.

However, our study still has several limitations. Although the ceRNA regulatory network is a significant mechanism of lncRNA‐ATB, we cannot exclude the possibility that lncRNA‐ATB promotes fibrogenesis via other molecular mechanisms, such as chromatin remodelling or lncRNA‐protein interaction. In this study, we focus on the function and underlying mechanism of lncRNA‐ATB during EMT, whereas its role in other cell types remains unclear. Similar to many other lncRNAs, lncRNA‐ATB is also poorly conserved across species. Therefore, we failed to verify its function in vivo further. At present, there are few studies on the underlying molecular mechanism by which TGF‐β1 promotes lncRNA‐ATB expression. Recent research suggests that Livin is an upstream regulator of lncRNA‐ATB, and it can stimulate TGF‐β1‐induced EMT by increasing lncRNA‐ATB in renal fibrosis.^37^ Nevertheless, detailed mechanisms in the regulatory process need further elucidation.

**FIGURE 7:**
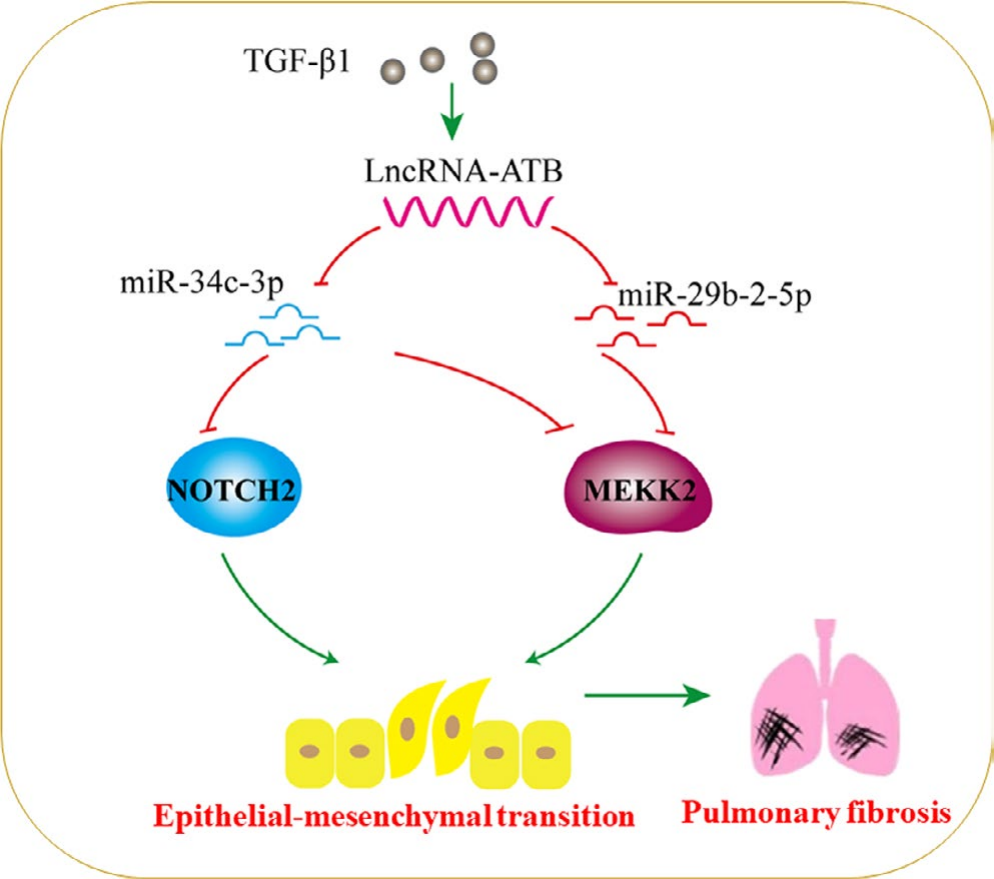
Schematic illustrations explain the signalling mechanisms by which lncRNA‐ATB regulates TGF‐β1‐induced EMT. LncRNA‐ATB acts as a sponge for miR‐29b‐2‐5p and miR‐34c‐3p, thereby promoting EMT and silica‐induced pulmonary fibrosis by regulating MEKK2 and NOTCH2

In conclusion, the data presented here identified lncRNA‐ATB as an essential determinant of TGF‐β1‐induced EMT and silica‐induced pulmonary fibrosis and ascribed its pro‐fibrotic effect to the regulation of MEKK2 and NOTCH2 signalling pathways via sponging miR‐29b‐2‐5p and miR‐34c‐3p (Figure 7). Strategies surrounding lncRNA‐ATB or its downstream miRNAs could represent a new effective therapeutic option to treat silicosis and other fibrotic diseases.

## CONFLICT OF INTEREST

The authors confirm that there are no conflicts of interest.

## AUTHOR CONTRIBUTIONS

**Qi Xu:** Conceptualization (equal); Investigation (equal); Methodology (equal); Project administration (equal); Writing‐original draft (equal). **Demin Cheng:** Investigation (equal); Methodology (equal). **Yi Liu:** Investigation (equal); Methodology (equal); Writing‐review & editing (equal). **Honghong Pan:** Investigation (equal). **Guanru Li:** Investigation (equal). **Ping Li:** Investigation (equal). **Yan Li:** Investigation (equal). **Wenqing Sun:** Investigation (equal). **Dongyu Ma:** Investigation (equal). **Chunhui Ni:** Conceptualization (equal); Funding acquisition (equal); Resources (equal); Supervision (equal); Writing‐review & editing (equal).

## Supporting information

Fig S1‐S5

## Data Availability

The data that support the findings of this study are available in the supplementary material of this article.
