# Supplementary material for: LncRNA‐ATB regulates epithelial‐mesenchymal transition progression in pulmonary fibrosis via sponging miR‐29b‐2‐5p and miR‐34c‐3p
Source: J Cell Mol Med. 2021 Jun 27;25(15):7294–306. doi: 10.1111/jcmm.16758 (PMC8335671; doi:10.1111/jcmm.16758)
Supplement: Supplementary file 1 — Fig S1‐S5 [file JCMM-25-7294-s001.pdf]

Supplementary information to:

LncRNA-ATB regulates epithelial-mesenchymal transition progression in pulmonary fibrosis via sponging miR-29b-2-5p and miR-34c-3p

Qi Xu[1], Demin Cheng[1], Yi Liu[1], Honghong Pan, Guanru Li, Ping Li, Yan Li, Wenqing Sun, Dongyu Ma, Chunhui Ni[*]

Center for Global Health, Key Laboratory of Modern Toxicology of Ministry of Education, School of Public Health, Nanjing Medical University, Nanjing 211166, China

[1]These authors contributed equally to this work and should be considered co-first authors.

*Corresponding author: Chunhui Ni, Center for Global Health, Key Laboratory of Modern Toxicology of Ministry of Education, Department of Occupational Medical and Environmental Health, School of Public Health, Nanjing Medical University, Nanjing 211166, China. E-mail: chni@njmu.edu.cn(Ch.-H. Ni),chninjmu@126.com(Ch.-H. Ni)

Key words: pulmonary fibrosis, EMT, lncRNA-ATB, ceRNA

Figure S1

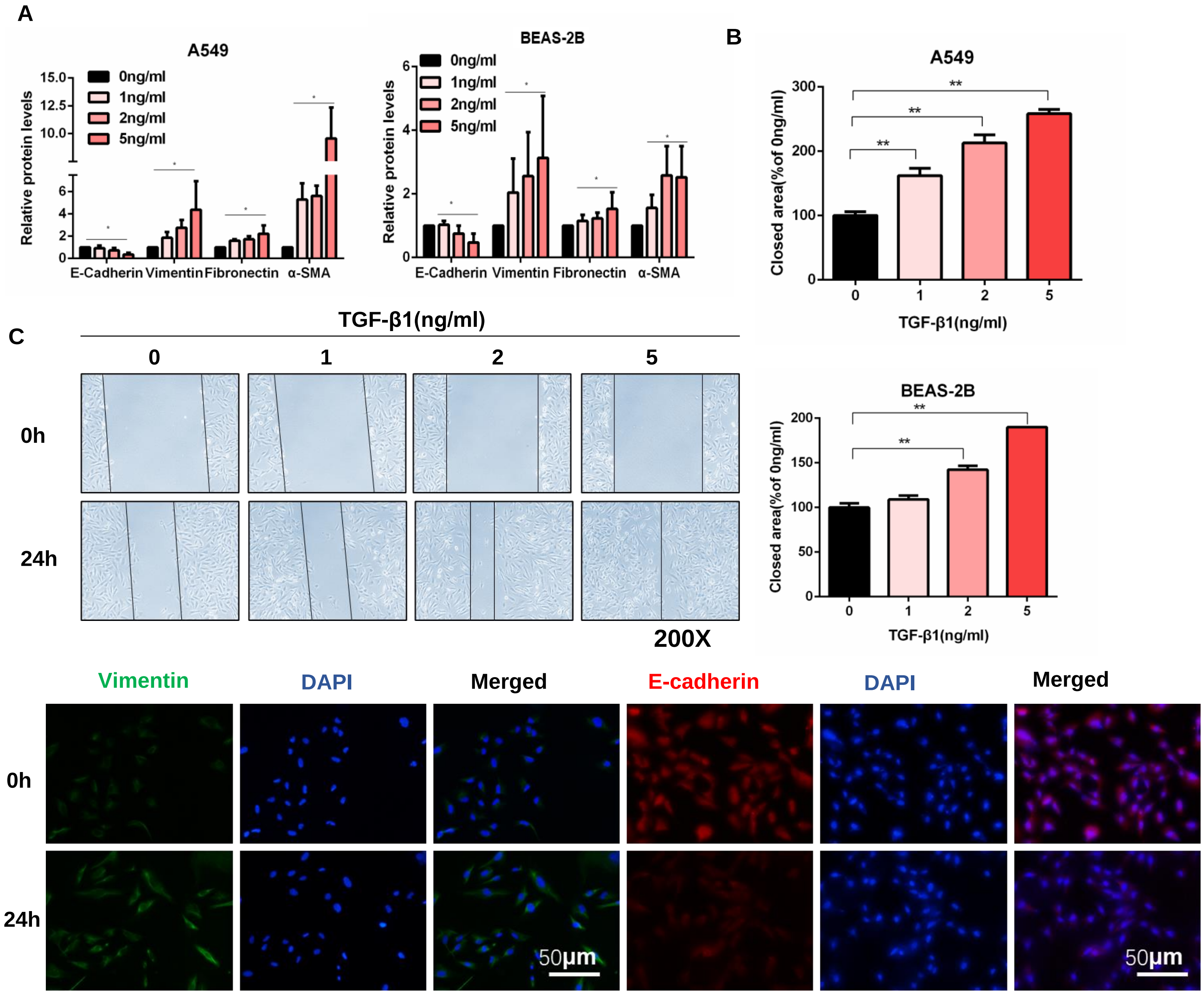
A
A549
Relative protein levels
15.0
12.5
10.0
6
4
2
0
0ng/ml
1ng/ml
2ng/ml
5ng/ml
E-Cadherin
Vimentin
Fibronectin
α-SMA
BEAS-2B
B
Closed area(%of 0ng/ml)
300
200
100
0
1
2
5
TGF-β1(ng/ml)
C
TGF-β1(ng/ml)
0h
24h
200X
150
50
Vimentin
DAPI
Merged
E-cadherin
50μm

Figure S2

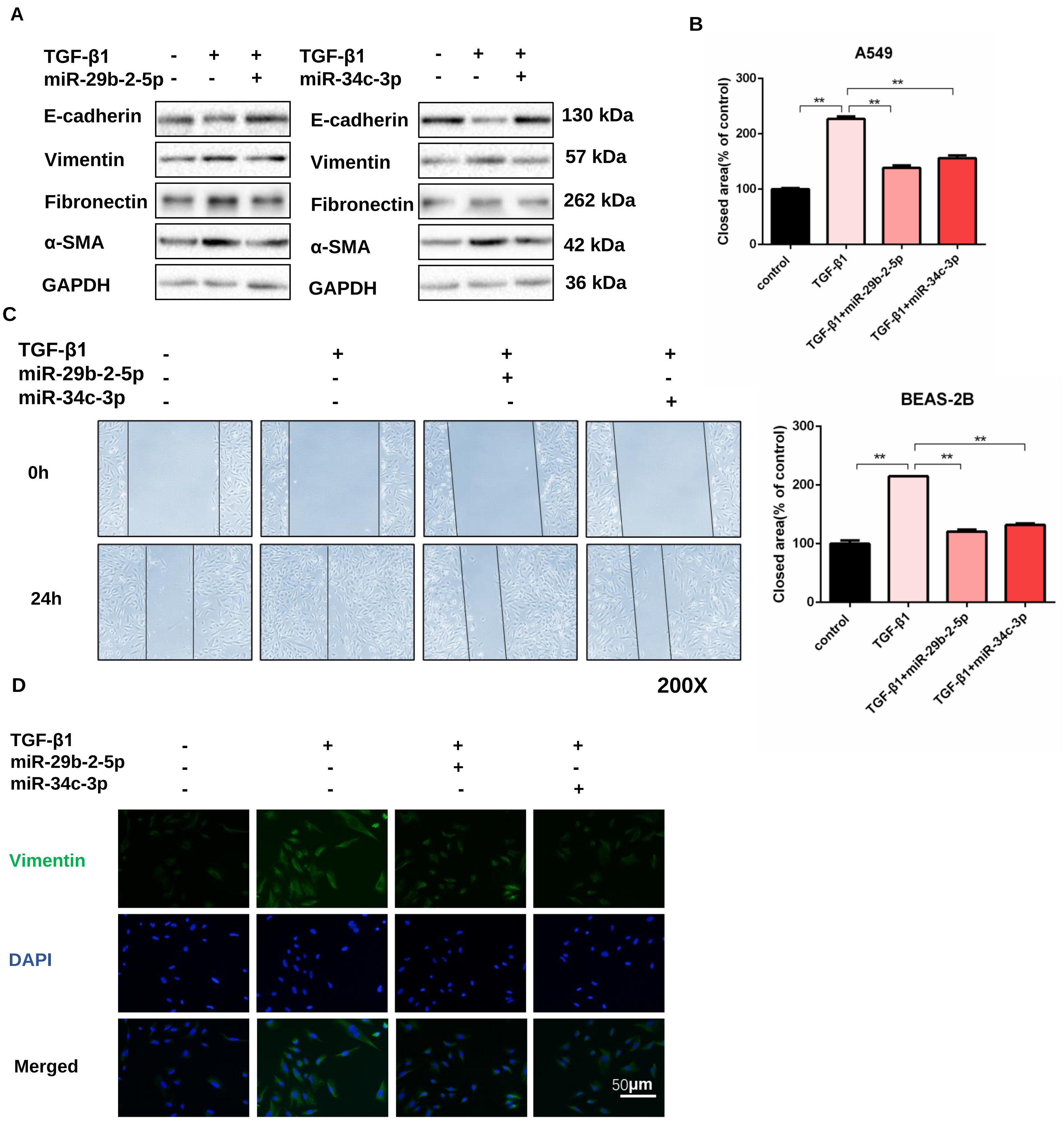
A
TGF-β1 - + +
miR-29b-2-5p - - +
TGF-β1 - + +
miR-34c-3p - - +
E-cadherin
Vimentin
Fibronectin
α-SMA
GAPDH
130 kDa
57 kDa
262 kDa
42 kDa
36 kDa
B
A549
Closed area(% of control)
300
200
100
0
control
TGF-β1
TGF-β1+miR-29b-2-5p
TGF-β1+miR-34c-3p
BEAS-2B
C
TGF-β1 - + + +
miR-29b-2-5p - - + -
miR-34c-3p - - - +
0h
24h
200X
D
TGF-β1 - + + +
miR-29b-2-5p - - + -
miR-34c-3p - - - +
Vimentin
DAPI
Merged
50μm

Figure S2

E

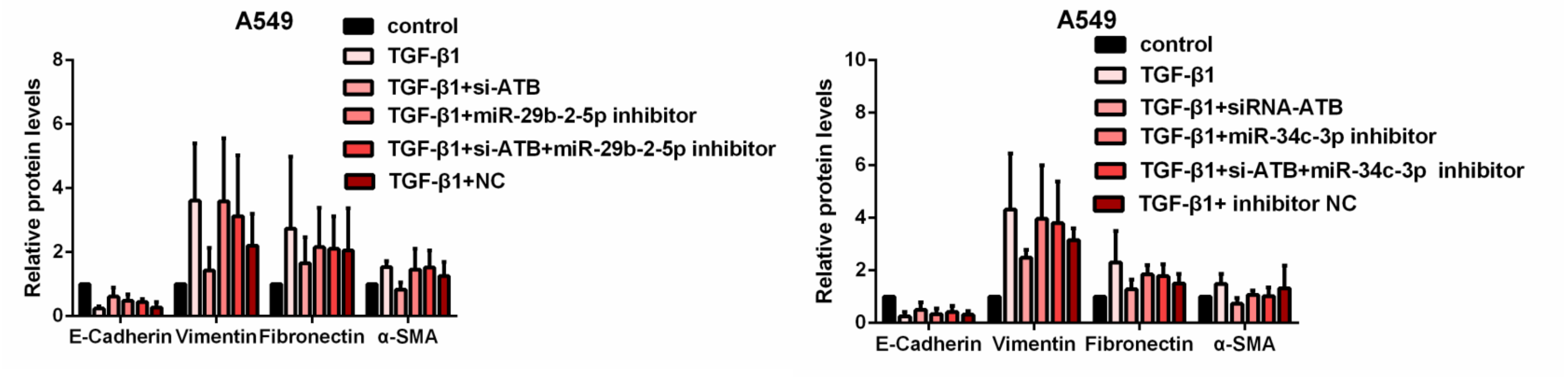

A549
control
TGF-β1
TGF-β1+si-ATB
TGF-β1+miR-29b-2-5p inhibitor
TGF-β1+si-ATB+miR-29b-2-5p inhibitor
TGF-β1+NC
Relative protein levels
0
2
4
6
8
E-Cadherin
Vimentin
Fibronectin
α-SMA
A549
control
TGF-β1
TGF-β1+siRNA-ATB
TGF-β1+miR-34c-3p inhibitor
TGF-β1+si-ATB+miR-34c-3p inhibitor
TGF-β1+ inhibitor NC
Relative protein levels
0
2
4
6
8
10
E-Cadherin
Vimentin
Fibronectin
α-SMA

F

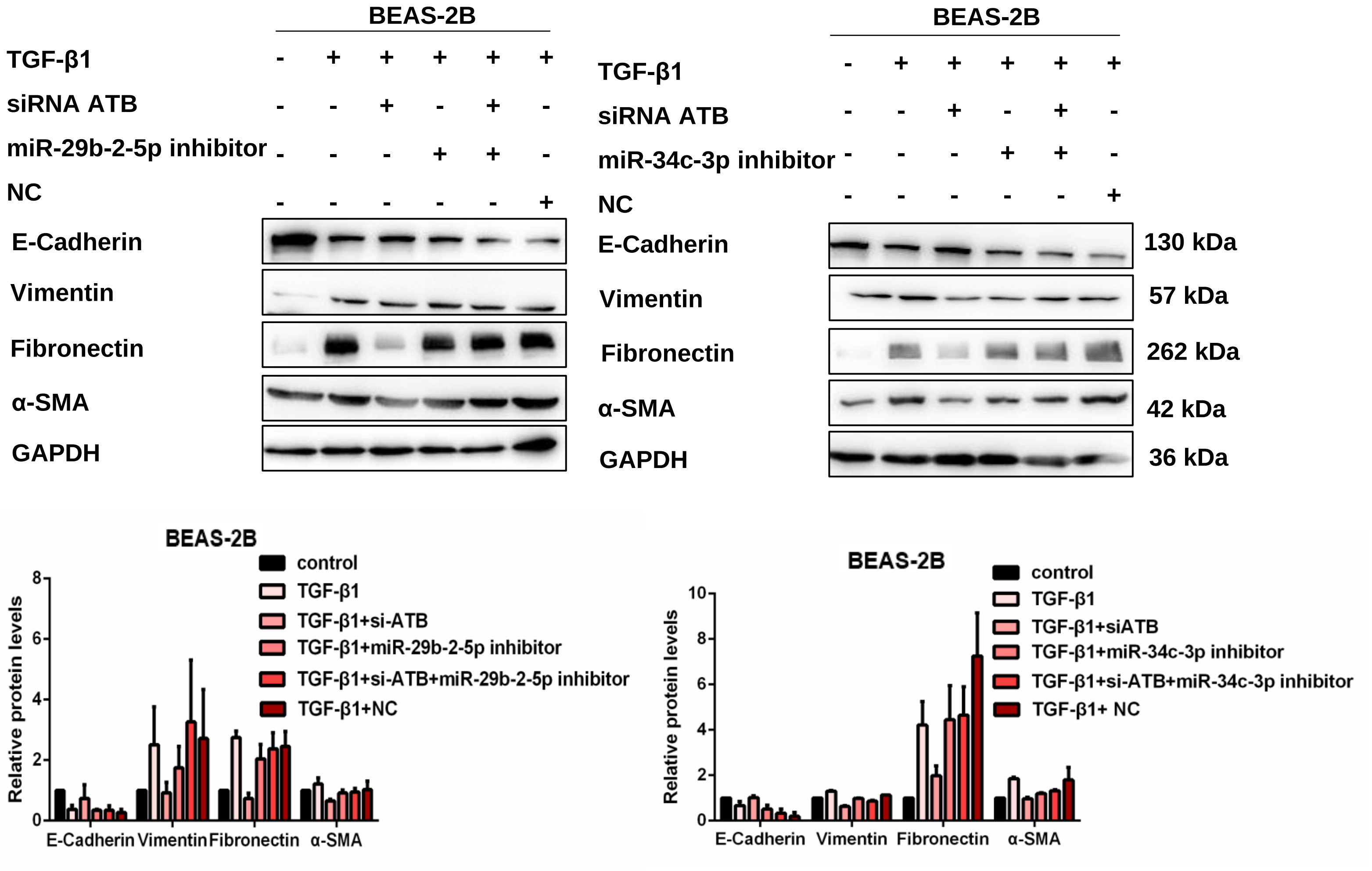

BEAS-2B
TGF-β1 - + + + + +
siRNA ATB - - + - + -
miR-29b-2-5p inhibitor - - - + + -
NC - - - - - +
E-Cadherin
Vimentin
Fibronectin
α-SMA
GAPDH
BEAS-2B
TGF-β1 - + + + + +
siRNA ATB - - + - + -
miR-34c-3p inhibitor - - - + + -
NC - - - - - +
E-Cadherin
Vimentin
Fibronectin
α-SMA
GAPDH
130 kDa
57 kDa
262 kDa
42 kDa
36 kDa
BEAS-2B
control
TGF-β1
TGF-β1+si-ATB
TGF-β1+miR-29b-2-5p inhibitor
TGF-β1+si-ATB+miR-29b-2-5p inhibitor
TGF-β1+NC
Relative protein levels
0
2
4
6
8
E-Cadherin
Vimentin
Fibronectin
α-SMA
BEAS-2B
control
TGF-β1
TGF-β1+siATB
TGF-β1+miR-34c-3p inhibitor
TGF-β1+si-ATB+miR-34c-3p inhibitor
TGF-β1+ NC
Relative protein levels
0
2
4
6
8
10
E-Cadherin
Vimentin
Fibronectin
α-SMA

Figure S3

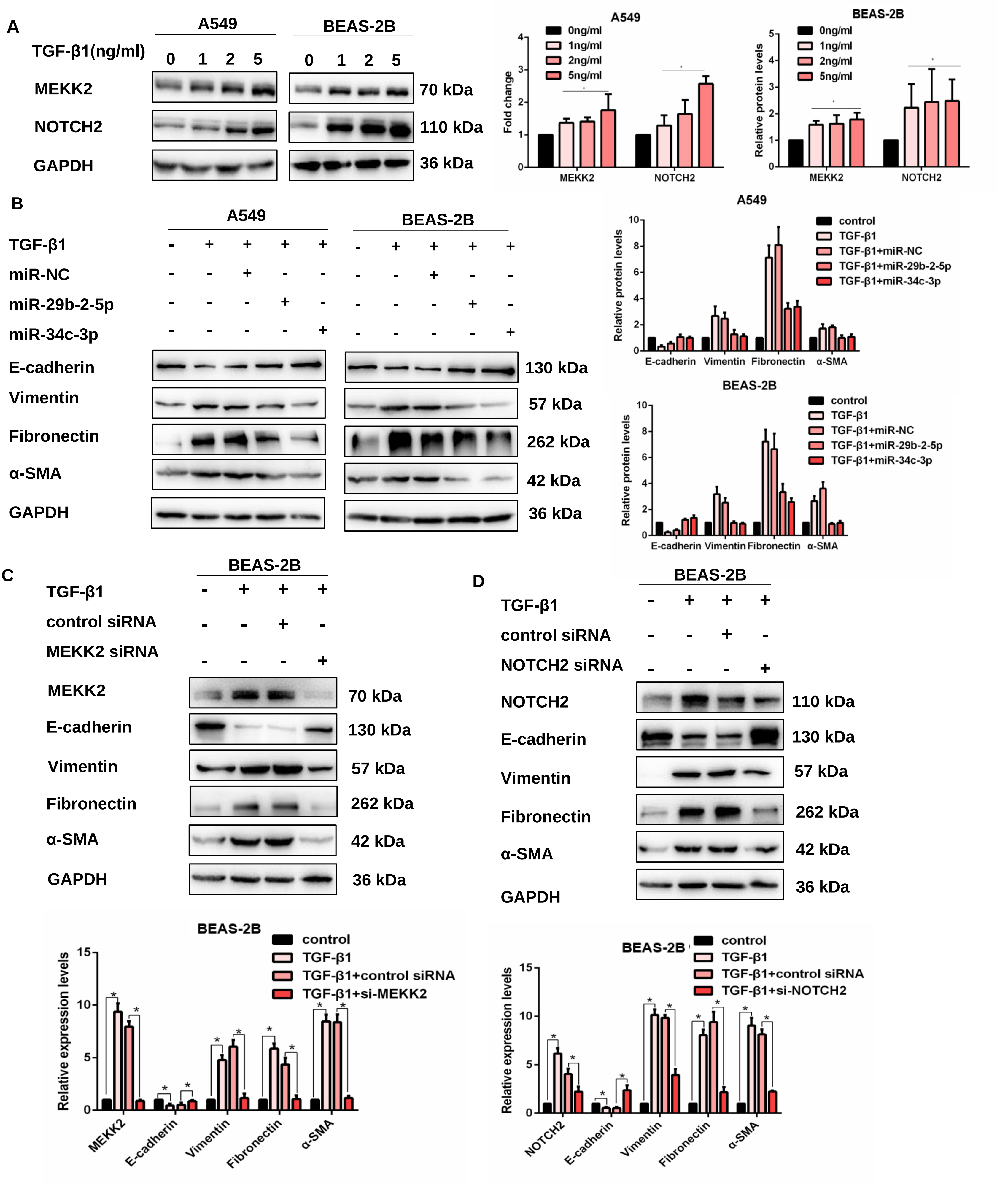
A
A549
BEAS-2B
TGF-β1(ng/ml) 0 1 2 5 0 1 2 5
MEKK2 70 kDa
NOTCH2 110 kDa
GAPDH 36 kDa
A549
0ng/ml
1ng/ml
2ng/ml
5ng/ml
Fold change
MEKK2
NOTCH2
BEAS-2B
Relative protein levels
B
A549
BEAS-2B
TGF-β1
miR-NC
miR-29b-2-5p
miR-34c-3p
E-cadherin 130 kDa
Vimentin 57 kDa
Fibronectin 262 kDa
α-SMA 42 kDa
GAPDH 36 kDa
control
TGF-β1
TGF-β1+miR-NC
TGF-β1+miR-29b-2-5p
TGF-β1+miR-34c-3p
C
BEAS-2B
TGF-β1
control siRNA
MEKK2 siRNA
MEKK2 70 kDa
E-cadherin 130 kDa
Vimentin 57 kDa
Fibronectin 262 kDa
α-SMA 42 kDa
GAPDH 36 kDa
Relative expression levels
TGF-β1+control siRNA
TGF-β1+si-MEKK2
D
BEAS-2B
NOTCH2 siRNA
NOTCH2 110 kDa
TGF-β1+si-NOTCH2

Figure S4

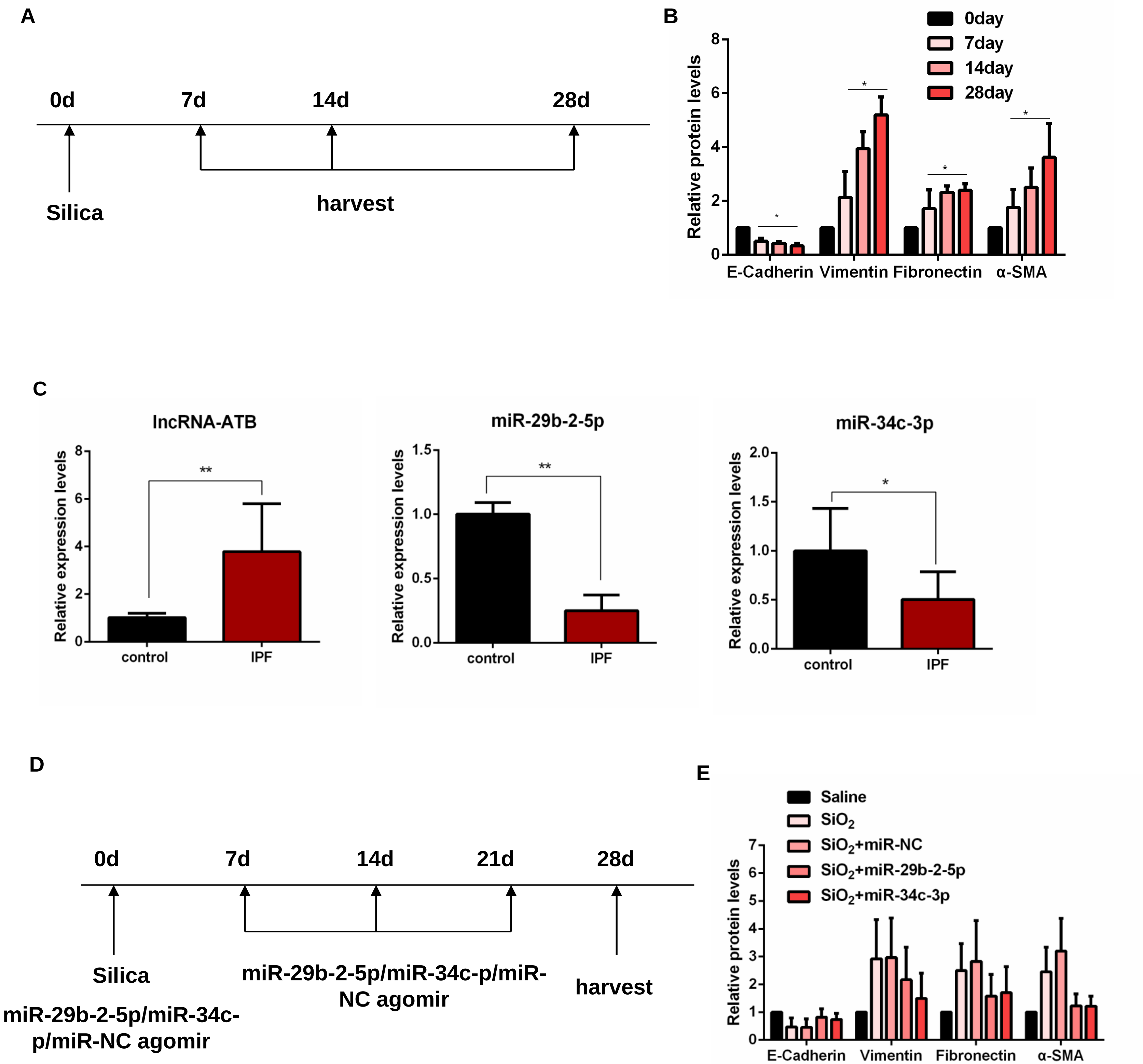

A
0d
7d
14d
28d
Silica
harvest
B
0day
7day
14day
28day
Relative protein levels
E-Cadherin
Vimentin
Fibronectin
α-SMA
C
lncRNA-ATB
miR-29b-2-5p
miR-34c-3p
Relative expression levels
control
IPF
D
0d
7d
14d
21d
28d
Silica
miR-29b-2-5p/miR-34c-p/miR-NC agomir
miR-29b-2-5p/miR-34c-p/miR-NC agomir
harvest
E
Saline
SiO2
SiO2+miR-NC
SiO2+miR-29b-2-5p
SiO2+miR-34c-3p
Relative protein levels
E-Cadherin
Vimentin
Fibronectin
α-SMA

Figure S5

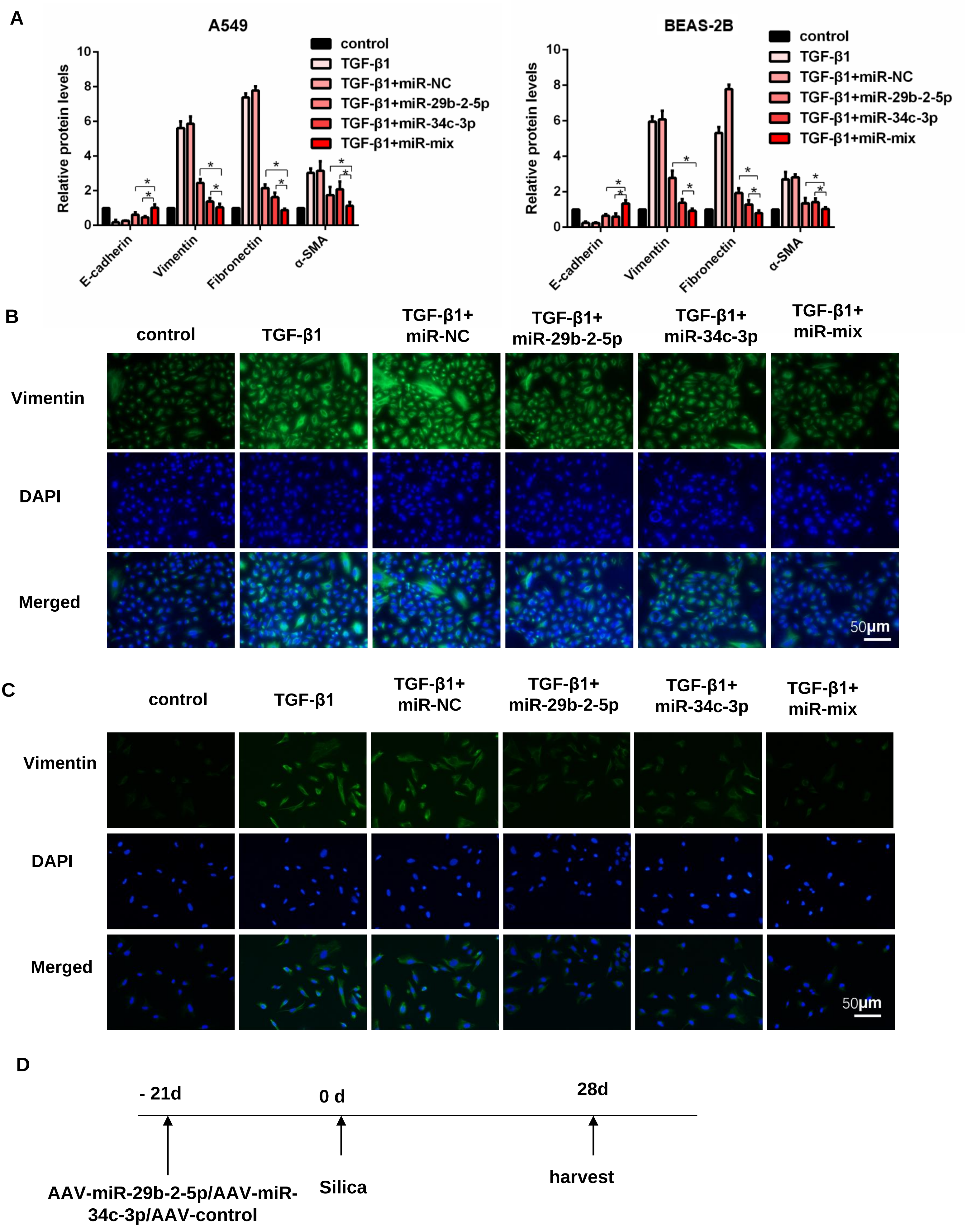
A
A549
BEAS-2B
Relative protein levels
control
TGF-β1
TGF-β1+miR-NC
TGF-β1+miR-29b-2-5p
TGF-β1+miR-34c-3p
TGF-β1+miR-mix
E-cadherin
Vimentin
Fibronectin
α-SMA
B
control
TGF-β1
TGF-β1+ miR-NC
TGF-β1+ miR-29b-2-5p
TGF-β1+ miR-34c-3p
TGF-β1+ miR-mix
Vimentin
DAPI
Merged
50μm
C
control
TGF-β1
TGF-β1+ miR-NC
TGF-β1+ miR-29b-2-5p
TGF-β1+ miR-34c-3p
TGF-β1+ miR-mix
Vimentin
DAPI
Merged
50μm
D
- 21d
0 d
28d
AAV-miR-29b-2-5p/AAV-miR-34c-3p/AAV-control
Silica
harvest

Figure S1. TGF-β1 promotes EMT process and decreases miR-29b-2-5p and miR-34c-3p levels. (A)Densitometric analysis of E-cadherin, Vimentin, Fibronectin and α-SMA in A549 and BEAS-2B cells were treated with 0, 1, 2, 5 ng/ml TGF-β1 for 48h (mean ± SD, n = 3), *P < 0.05 difference from untreated cells. (B) Wound healing assays were evaluated by the closed areas by migrated cells (%) in A549 cells (mean ± SD, n = 3), **P< 0.01. (C) Wound healing assays were performed and evaluated by the closed areas by migrated cells (%) in BEAS-2B cells (mean ± SD, n = 3), **P< 0.01. (D) Immunofluorescence staining of Vimentin and E-cadherin in BEAS-2B cells for the control and TGF-β1 (5ng/ml) treatment groups. Green represents Vimentin staining; Red represents E-cadherin staining; blue represents nuclear DNA staining by DAPI. The scale bar is 50μm.

Figure S2. MiR-29b-2-5p and miR-34c-3p mediate the function of lncRNA-ATB in regulating EMT.
(A) Western blot detected levels of E-cadherin, Vimentin, Fibronectin, and α-SMA in BEAS-2B cells transfected with miR-29b-2-5p or miR-34c-3p mimic then treated with 5ng/ml TGF-β1 for 48h. (B) Wound healing assays were evaluated by the closed areas by migrated cells (%) in A549 cells (mean ± SD, n = 3), **P< 0.01. (C) Wound healing assays were performed and evaluated by the closed areas by migrated cells (%) in BEAS-2B cells (mean ± SD, n = 3), **P< 0.01. (D) The expression of Vimentin was detected by immunofluorescence staining, in BEAS-2B cells transfected with miR-29b-2-5p or miR-34c-3p mimic then treated with 5ng/ml TGF-β1 for 48h. (E) Densitometric analysis of E-cadherin, Vimentin, Fibronectin and α-SMA in A549 cells for the indicated groups. (F) Western blot and densitometric analysis of the protein expression of E-cadherin, Vimentin, Fibronectin, and α-SMA in treated BEAS-2B cells for the indicated groups.

Figure S3.MEKK2 and NOTCH2 are two functional downstream targets of miR-29b-2-5p and miR-34c-3p. (A) Western blot and densitometric analysis of the protein expression of E-cadherin, Vimentin, Fibronectin and α-SMA in A549 and BEAS-2B cells were treated with 0, 1, 2, 5 ng/ml TGF-β1 for 48h (mean ± SD, n = 3), *P < 0.05 difference from untreated cells. (B) Western blot and densitometric analysis of the protein expression of E-cadherin, Vimentin, Fibronectin and α-SMA in A549 and BEAS-2B cells for the indicated group. (C) Western blot and densitometric analysis of MEKK2, NOTCH2, E-cadherin, Vimentin, Fibronectin and α-SMA in BEAS-2B cells transfected with MEKK2 siRNA or its negative control then treated with 5ng/ml TGF-β1 for 48h. (D) Western blot and densitometric analysis of NOTCH2, E-cadherin, Vimentin, Fibronectin and α-SMA in BEAS-2B cells transfected with NOTCH2 siRNA or its negative control then treated with 5ng/ml TGF-β1 for 48h.

Figure S4. MiR-29b-2-5p or miR-34c-3p accelerates silica-induced pulmonary fibrosis resolution by regulating EMT. (A) Diagram shows the mouse treatment timeline. The C57BL/6 mice were sacrificed on days 7, 14, and 28 after intratracheal instillation of silica suspended saline and saline. (B) Densitometric analysis of the protein expression of E-cadherin, Vimentin, Fibronectin, and α-SMA in mouse lung tissues. (C) The expression of lncRNA-ATB, miR-29b-2-5p and miR-34c-3p in lung tissues of control and IPF patients was detected by qRT-PCR(mean ± SD, $n = 3$), $^*P < 0.05$, $^{**}P < 0.01$. (D) Diagram shows the mouse model of miR-29b-2-5p and miR-34c-3p overexpression in silica-induced mouse pulmonary fibrosis. The C57BL/6 mice were co-transfected miR-29b-2-5p, miR-34c-3p or miR-NC agomir with silica suspension via intratracheal instillation, and the mice were injected with miR-29b-2-5p, miR-34c-3p or miR-NC agomir via the tail vein on day 7, 14 and 21. Then the tissues were harvested on day 28 ($n = 8$ for each group). (E) Densitometric analysis of the protein expression of E-cadherin, Vimentin, Fibronectin and α-SMA in mouse lung tissues treated with miR-29b-2-5p or miR-34c-3p agomir for 28 days were determined by western blot and found decreased compared with the silica+ miR-NC agomir group.

Figure S5. Combination of miR-29b-2-5p and miR-34c-3p exerts a synergic effect on EMT and silica-induced pulmonary fibrosis. (A) Densitometric analysis of the protein expression of E-cadherin, Vimentin, Fibronectin, and α-SMA in A549 and BEAS-2B cells for the indicated groups (mean ± SD, $n = 3$), $^*P < 0.05$. (B) Immunofluorescence staining of Vimentin in A549 cells. Green represents Vimentin staining; blue represents nuclear DNA staining by DAPI. The scale bar is 50μm. (C) Immunofluorescence staining of Vimentin in BEAS-2B cells. Green represents Vimentin staining; blue represents nuclear DNA staining by DAPI. The scale bar is 50μm. (D) Diagram shows the experimental design. Mice were administered with AAV-miR-29b-2-5p, AAV-miR-34c-3p, or AAV-NC intratracheally at a dose of 1×10^{11} vectors in a total of 0.05ml of sterile saline. Three weeks later, mice were treated with 50mg/kg SiO_2, and the control group was treated with 0.05ml of sterile saline using the same method for four weeks.
